# Membrane phospholipids control gating of the mechanosensitive potassium leak channel TREK1

**DOI:** 10.1038/s41467-023-36765-w

**Published:** 2023-02-25

**Authors:** Philipp A. M. Schmidpeter, John T. Petroff, Leila Khajoueinejad, Aboubacar Wague, Cheryl Frankfater, Wayland W. L. Cheng, Crina M. Nimigean, Paul M. Riegelhaupt

**Affiliations:** 1 Department of Anesthesiology, Weill Cornell Medical College, New York, NY USA; 2 Department of Anesthesiology, Washington University School of Medicine in St. Louis, St. Louis, MO USA; 3 Department of Endocrinology, Metabolism, and Lipid Research, Washington University School of Medicine in St. Louis, St. Louis, MO USA; 4 Department of Physiology and Biophysics, Weill Cornell Medical College, New York, NY USA; 5 Department of Biochemistry, Weill Cornell Medical College, New York, NY USA

**Keywords:** Potassium channels, Cryoelectron microscopy, Ion transport

## Abstract

Tandem pore domain (K2P) potassium channels modulate resting membrane potentials and shape cellular excitability. For the mechanosensitive subfamily of K2Ps, the composition of phospholipids within the bilayer strongly influences channel activity. To examine the molecular details of K2P lipid modulation, we solved cryo-EM structures of the TREK1 K2P channel bound to either the anionic lipid phosphatidic acid (PA) or the zwitterionic lipid phosphatidylethanolamine (PE). At the extracellular face of TREK1, a PA lipid inserts its hydrocarbon tail into a pocket behind the selectivity filter, causing a structural rearrangement that recapitulates mutations and pharmacology known to activate TREK1. At the cytoplasmic face, PA and PE lipids compete to modulate the conformation of the TREK1 TM4 gating helix. Our findings demonstrate two distinct pathways by which anionic lipids enhance TREK1 activity and provide a framework for a model that integrates lipid gating with the effects of other mechanosensitive K2P modulators.

## Abstract (web summary)

Tandem pore (K2P) potassium channels set the cellular resting membrane potential in tissues throughout the body. Here, authors show how the composition of phospholipid within the bilayer may directly alter gating in this family of ion channels.

## Introduction

Tandem pore (K2P) potassium ion channels play vital roles in human physiology, chiefly responsible for maintaining cellular resting membrane potential. Modulation of K2P channel activity influences cellular excitability, altering the degree of input signal required to reach action potential thresholds^1^. For the mechanosensitive subfamily of K2Ps (including the TREK1, TREK2, and TRAAK channels), diverse stimuli control channel activity, including lipids, pH, heat, membrane tension, post-translational modification, and an array of pharmacologically active agents including volatile anesthetics, antidepressants, and neuroprotective agents^2–4^. While many of these dissimilar K2P gating cues seem to share overlapping mechanisms of action, a unifying explanation describing how these diverse and biophysically distinct signals become integrated by the channel architecture remains incomplete.

Two key regions of the mechanosensitive K2P channel structure appear to control channel function, the conformationally flexible TM4 helix, and the selectivity filter. Movement of TM4 is the most significant conformational rearrangement found in crystallographic studies of K2Ps^5–7^, with this helix positioned in either a TM4 “up” or TM4 “down” state. TM4 movement is believed to have two distinct consequences. First, the position of TM4 is thought to modulate potassium ion permeability at the selectivity filter^8–14^, by a mechanism akin to the C-type gating behavior found in other potassium channels^15–17^. For many K2Ps, this coupling between TM4 position and selectivity filter behavior is complex, with both the “up” and “down” TM4 conformations capable of supporting conductive channel states^6,7,13,18^, albeit with differing voltage dependencies and pharmacological sensitivities^13,19^. A secondary consequence of TM4 helix movement is the appearance of a lateral fenestration to the lipid bilayer when TM4 is in the “down” state, suggesting the possibility that membrane phospholipids might enter the channel pore to block conduction^7,12^. This assertion is supported by several crystallographic structures of K2Ps in the TM4 “down” state, where electron density is found adjacent to the open lateral fenestration and below the selectivity filter, occluding the ion conduction pathway^7,12,20^.

Whereas pore block by membrane phospholipids is predicted to inhibit K2P channel activity, enrichment of the membrane environment with phosphatidic acid (PA) or other anionic lipids potentiate mechanosensitive K2P channel activity^21,22^. The activating effect of PA on TREK1 is of particular biological relevance, as phospholipase D2 (PLD2) binds to the C-terminus of TREK1^23^ and serves as an inducible local source of PA production in vivo. The positive modulatory influences of anionic lipids and membrane tension appear to be mechanistically intertwined^24–26^ and both mechanical force and volatile anesthetics have been found to disrupt the association between TREK1 and PLD2^27^. While mixed positive and negative modulatory effects of lipids have been reported across the K2P superfamily^28–31^, the molecular details describing how lipids bind and interact with K2Ps to modulate their function have not been clearly defined.

In this study, we explore the molecular basis for phospholipid modulation in the mechanosensitive K2P channel TREK1, combining single particle cryo-EM, native mass spectrometry (MS) and MS-based lipidomics, and ion flux-based functional studies. We solve cryo-EM structures of apo TREK1 (3.2 Å) as well as TREK1 bound to the anionic lipid POPA (2.8 Å) or the zwitterionic lipid POPE (3.2 Å). Each condition yielded a distinct conformation of the TREK1 channel, featuring TM4 helices in the “TM4 up” (POPA condition), “TM4 down” (POPE condition), and a previously undescribed asymmetric “TM4 up/down” conformation in the absence of exogenously added lipids. We characterize distinct binding sites for both POPA and POPE that explain the divergent effects of these lipids on TREK1 conformation and function. Our results elucidate multiple interdependent pathways by which membrane phospholipids modulate K2P activity to provide the basis for a comprehensive TREK1 gating model that accounts for the convergence of phospholipid regulation with mechanical stretch and many other K2P gating modalities.

## Results

### Apo TREK1 is an asymmetric dimer

**Fig. 1:**
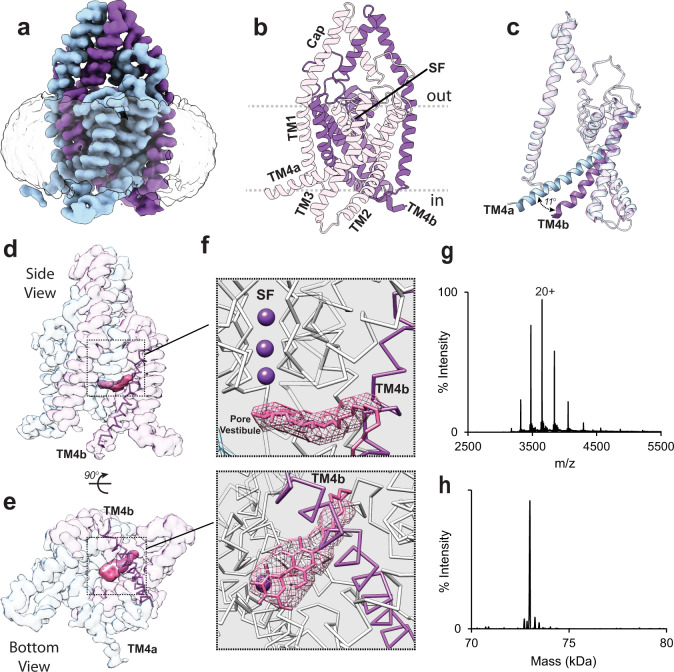
Subunit asymmetry in the TREK1 apo cryo-EM structure. **a** Cryo-EM density map of the apo state of the TREK1 channel and **b** a structural model of the channel, with the potassium ion selectivity filter (SF), extracellular cap domain, and four transmembrane domains labeled. **c** A superposition of the two apo TREK1 subunits, showing asymmetric positioning of TM4a (blue) relative to TM4b (purple), due to an 11° tilt in the distal portion of TM4. Transparent views of the TREK1 cryo-EM density map **d** side view and **e** bottom view, with density outside of the TREK1 map near the TM4b helix highlighted (pink). Magnified views (**f**), showing that this central density occludes the pore, sterically prevents TM4b from moving into the “up” state, and can be well modeled by a molecule of DDM (also shown in Supplementary Fig. 4). DDM density derived from the final unsharpened TREK1 apo map, visualized at a contour threshold of 0.0065 (**g**). Native mass spectrum of TREK1 dimer (20+ charge state is labeled), with deconvoluted spectrum (**h**), showing no evidence of phospholipid bound to TREK1.

Using single particle cryo-EM, we first solved a 3.27 Å resolution structure of TREK1 solubilized in DDM detergent in an unexpected conformation (Fig. 1 and Supplementary Fig. 5). Overall, this cryo-EM structure exhibits K2P architectural features consistent with prior crystallographic studies^32–34^: a dimeric channel assembly with each subunit composed of four transmembrane helices, a domain-swapped cap located above the selectivity filter, and an extended membrane facing TM2/TM3 loop within each subunit (Fig. 1a, b). However, unlike all prior TREK1 structures, where intermolecular crystallization contacts immobilize the TM4 helix in an “up” conformation, the TM4s in our cryo-EM samples are unconstrained prior to sample vitrification. As a result, we find that TREK1 exhibits a previously unobserved asymmetric TM4 “up/down” conformational state. This asymmetry is due to an ~11° bend in the TM4a helix in the “up” state relative to the TM4b helix in the “down” state (Fig. 1c). While prior structures of TRAAK channels also exhibited asymmetric positioning of the TM4 helices^5,7,12^, this asymmetry has always been attributed to crystallization artifacts and has not been considered biologically relevant. By contrast, the clear TM4 asymmetry in our TREK1 cryo-EM structure suggests the possibility that the two K2P subunits are not tightly coupled and are able to move independently.

The apparent cause of the observed TREK1 TM4 asymmetry is the presence of a density non-contiguous with the TREK1 density map but near the TM4b helix, sterically impeding TM4b from adopting the “up” conformation (Fig. 1d–f, pink). This density extends directly below the TREK1 selectivity filter, occluding the ion permeation pathway (Fig. 1f). As lipids have previously been suggested to occupy the open fenestration in TRAAK K2P channels^7,12^ and the density observed in our TREK1 structure mirrors these prior observations, we attempted to determine if this observed density is in fact a phospholipid co-purified from the yeast cell membrane. Native MS analyses of DDM-purified TREK1 showed intact TREK1 dimer with a charge distribution similar to results previously observed for TRAAK in DM detergent^35^, but did not identify any lipids bound to the TREK1 protein (Fig. 1g, h). To exclude the possibility that co-purified lipid might only be loosely associated with TREK1 and could be lost during sample preparation or ionization for native MS analysis, we performed a Bligh-Dyer extraction of the purified TREK1 sample and analyzed the extracted organic phase by MS. Here we again found no evidence of phospholipids or sterols co-purified with the TREK1 protein, despite obtaining robust signals from phospholipid or cholesterol standards (Supplementary Fig. 2 and Supplementary Table 2).

The TREK1 protein is solubilized in *n*-dodecyl-β-d-maltoside (DDM) detergent and a molecule of DDM easily fits the observed density if the DDM is oriented with its maltoside sugar moieties located within the pore and hydrocarbon tail projecting outward toward the fenestration (Fig. 1c–e and Supplementary Fig. 5e). While the positioning of this pore-bound DDM molecule is inverted relative to the orientation of DDM molecules in the micelle surrounding the TREK1 protein, the presence of a detergent molecule within the TREK1 pore suggested that this site could be a target for lipids in a biological membrane. Utilizing our DDM solubilized apo TREK1 protein preparation as an experimentally verified phospholipid- and sterol-free starting point, we added back chemically distinct phospholipid species to the TREK1 protein and assessed for changes in channel function and structure.

### Modulatory phospholipids alter TREK1 function and structure

**Fig. 2:**
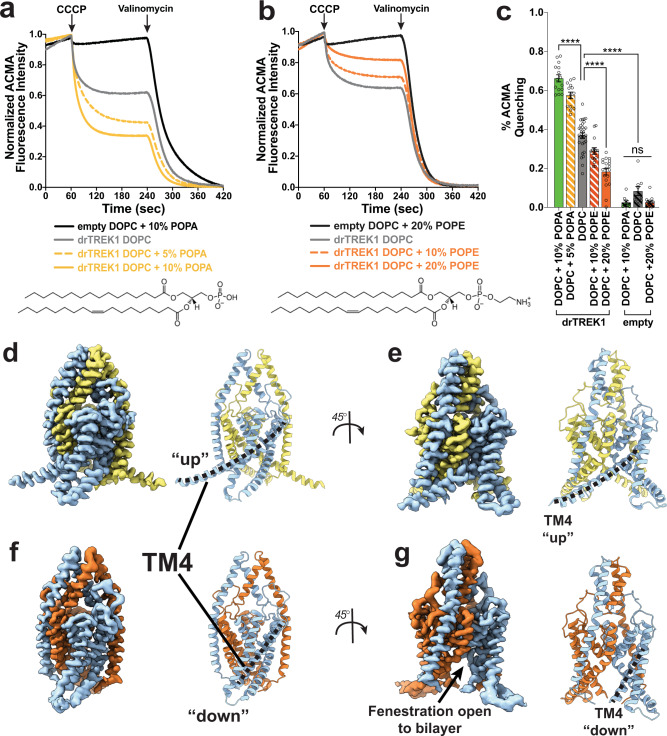
Functional and structural effects of phospholipids on the TREK1 channel. ACMA fluorescence quenching assays to study the function of purified TREK1 protein in varied lipid compositions (**a**–**c**). Quenching was initiated by the addition of the proton ionophore CCCP and allowed to proceed to completion by the addition of the potassium ionophore valinomycin. Signals were normalized to the final baseline value prior to the addition of CCCP (time *t* = 59 s) and the final fluorescence value in the tracings (time *t* = 420 s). Averaged composite traces of all quenching reaction measurements are shown in (**a**) and (**b**), with liposome compositions indicated. TREK1-dependent ACMA quenching was measured as (1 - Normalized ACMA fluorescence intensity at time *t* = 239 s) and is shown in (**c**). Data values for all individual measurements are shown and bars represent mean ± SEM. For all conditions, *n* = 11–31 independent measurements derived from 2–7 separate proteoliposome reconstitutions. Source data are available as a source data file. Statistical analysis was performed by one-way ANOVA with a Dunnett’s multiple comparison test with results indicated, ns not significant, ****p < 0.0005. **d**–**f** Cryo-EM density maps and matching structural models of TREK1 channels in DDM micelles supplemented with POPA (**d**, **e**) or POPE (**f**, **g**). In the POPA structure, both TM4 are in the “up” conformation (**d**) and the membrane facing fenestration is closed (**e**). In the presence of POPE, both TM4 are in the “down” conformation (**f**) and the membrane facing fenestration is open (**g**).

Mechanosensitive K2Ps have previously been found to respond to changes in the composition of the surrounding phospholipid bilayer^21,23,35^. We first determined that our purified zebrafish TREK1 protein construct is similarly modulated by phospholipids, utilizing an ACMA-based fluorescence quenching assay as a readout of ion flux through the TREK1 channel. For these experiments, we reconstituted TREK1 into pure 1,2-dioleoyl phosphatidylcholine (DOPC) liposomes and demonstrated that ACMA quenching increases in a TREK1 concentration-dependent manner (Supplementary Fig. 1b, c) but is absent in empty liposomes lacking TREK1. We then held the reconstituted TREK1 protein concentration constant and altered the phospholipid composition of the proteoliposomes, demonstrating that the anionic lipid 1-palmitoyl-2-oleoyl PA (POPA) causes a dose-dependent increase in TREK1 activity while the zwitterionic lipid 1-palmitoyl-2-oleoyl PE (POPE) inhibits TREK1 function (Fig. 2a–c). As a control, we ensured that alterations in proteoliposome lipid composition had no effect on either the incorporation efficiency of a C-terminally GFP-tagged TREK1 protein (as measured by GFP fluorescence after SDS-PAGE, Supplementary Fig. 1d) or the relative orientation of the TREK1 protein within the liposomes (as measured by accessibility to 3 C protease cleavage of the attached GFP, Supplementary Fig. 1e, f), such that measured differences in ACMA quenching in the varied lipid compositions are best explained by changes in TREK1 channel activity.

We next sought to define the structural basis for the observed influence of phospholipids on TREK1 function. For this, we dissolved POPA or POPE phospholipids in DDM detergent to form mixed lipid/detergent micelles and incubated these mixed micelles with DDM solubilized TREK1 protein. As TREK1 is mechanosensitive, we specifically chose this approach over the use of lipid nanodiscs, attempting to directly probe the influence of lipids and avoid introducing a component of membrane tension that might accompany the nanodisc approach. We solved two additional structures of TREK1 in the presence of either POPA or POPE lipids (at 2.82 and 3.27 Å resolutions, respectively: Fig. 2 and Supplementary Figs. 7–9). Whereas the apo TREK1 structure featured asymmetric positioning of the TREK1 TM4 helices, the experimentally added lipids induced TREK1 symmetry, with the TREK1 POPA structure in the TM4 “up” conformation and the TREK1 POPE structure in the TM4 “down” conformation.

Selectivity filter asymmetry has been proposed to play a role in the gating of TREK1 channels^34^ and our results suggest the intriguing possibility that symmetry/asymmetry transitions within the transmembrane region of K2Ps may also play a role in K2P function. In our structures, conformational changes in the TM4 helices did not lead to conformational rearrangements of the selectivity filter regions (Supplementary Fig. 3d, e), as have been observed to occur after TREK1 protein crystals are soaked in low concentrations of potassium^34^. We find that selectivity filter behavior in our three cryo-EM structures mirrors results obtained from structures of TREK2^6^, with the TM4 “up” state showing full filter occupancy and the TM4 “down” and asymmetric TM4 “up/down” structures showing ion occupancy at S2-S4, with an empty S1 site (Supplementary Fig. 3f–h). However, our structural studies were performed in solutions containing 150 mM potassium, a condition that is likely to stabilize the K2P selectivity filter gate. It remains possible that TM4 movements modulate the TREK1 selectivity filter structure when the outward face of the channel is in a physiological low potassium environment, one we have not captured in these studies.

Upon comparing the TREK1 structures determined in either the absence or presence of exogenous POPA or POPE, we find that the asymmetric subunits in the apo TREK1 structure superimpose nearly identically on the appropriately matched subunit from the symmetric structures, with negligible differences in all-atom root mean square deviation (RMSD) between the matched subunits (Supplementary Fig. 3a–c). Whereas the protein chains are nearly identical, several well-defined cryo-EM densities that appear to be phospholipid molecules are readily apparent in the structures obtained in the presence of experimentally introduced phospholipids but absent in the apo TREK1 structure. Prior functional and native MS^21,35^ studies of mechanosensitive K2Ps have suggested that PA and PE lipids bind at distinct channel sites and our structures confirm this, as the identified phospholipid densities in the two lipid conditions do not overlap. These lipid densities and their binding sites on the TREK1 protein provide a molecular basis for the divergent effects of these distinct phospholipids on TREK1 function and structure.

### POPA lipids bind TREK1 at multiple sites

**Fig. 3:**
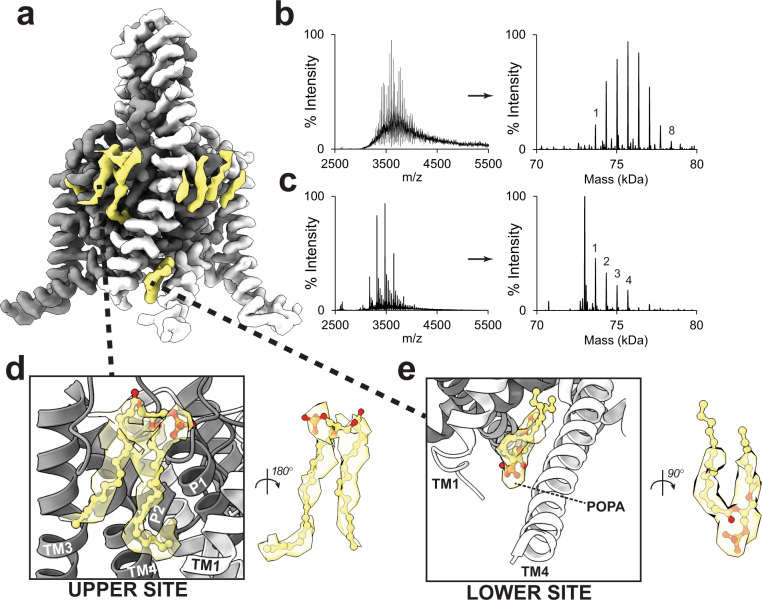
Identification of POPA lipid binding sites in the TREK1 channel structure. Cryo-EM density map of TREK1 solubilized in DDM detergent supplemented with POPA lipids (**a**), highlighting locations of bound POPA molecules (yellow). (**b**) Native mass spectrum of TREK1 dimer with POPA (left), and deconvoluted spectrum (right) showing multiple peaks corresponding to TREK1 dimer with up to eight bound POPA molecules (numbers above peaks indicate the number of bound POPA). Under more activating MS conditions (**c**), the number of bound POPA lipids is reduced to four, suggestive of two relatively higher affinity binding sites per subunit in the TREK1 dimer. **d**, **e** Zoomed-in views of two POPA binding sites identified in the cryo-EM density map where the POPA lipid interacts with the core of the TREK1 protein. Visualized lipid densities are derived from the final unsharpened C2 symmetrized TREK1 POPA map, visualized at a contour threshold of 0.0086.

**Fig. 4:**
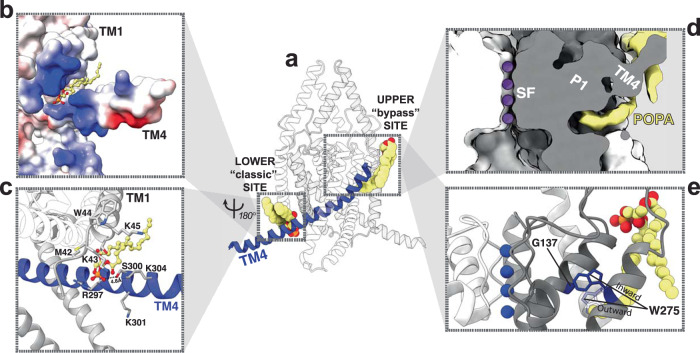
Molecular details of the POPA lipids binding sites. The upper and lower POPA binding sites identified in the cryo-EM density map flank the TM4 helix (**a**). At the lower site (**b**, **c**), the well-resolved POPA headgroup sits in a groove between TM1 and TM4. A coulombic potential surface representation of the lower site (**b**) with a molecular representation shown in (**c**), demonstrates the strong electropositive nature of the lower binding site. At the upper site (**d**, **e**), the cryo-EM density for a lipid acyl tail can be seen inserting itself underneath TM4 to sit behind the selectivity filter pore helices. A molecular representation of this lipid binding site (**e**) shows that the lipid tail displaces the W275 residue from its outward-facing position in the TM4 down state (transparent) to an inward-facing orientation, bringing W275 close to the G137 residue.

The cryo-EM structure of TREK1 in POPA shows evidence for numerous densities consistent with bound POPA lipids (Fig. 3a). Given this structural observation, we repeated native MS studies of TREK1 in the presence of the same concentration of POPA used for cryo-EM. In two replicate experiments, we found evidence for up to 8 (repeat 1, Fig. 3b) or 12 (repeat2, Supplementary Fig. 2f, g) POPA lipids bound to the TREK1 protein dimer, suggesting the possibility of 4 to 6 sites per subunit where POPA lipids might bind in the dimeric channel architecture. In both replicate experiments, increasing the activation energy in the mass spectrometer to the maximal level where a TREK1 dimer signal is still present led to the loss of all but four of the bound POPA molecules (Fig. 3c and Supplementary Fig. 2), suggestive of two tightly bound POPA molecules per subunit in the dimeric channel. This result matched well with our TREK1 POPA structure. There are two POPA binding sites where the lipid density interacts directly with the core of the protein (Fig. 3d, e). These lipid densities are the strongest and most complete in the TREK1 POPA structure and were the first lipids to become apparent during data processing, remaining well-defined even in unsharpened cryo-EM maps. These two sites envelop the TM4 helix, with one site at the extracellular face of the channel near the top of TM4 and the other at the C-terminal end of TM4 near the cytoplasmic face of TREK1 (Fig. 4a). The remainder of the structurally identified lipid densities encircle the extracellular face of the TREK1 transmembrane domains, presumably as more loosely associated annular lipids (Fig. 3a).

### A POPA lipid tail interacts with functionally critical residues behind the selectivity filter

At the upper POPA binding site, an acyl tail of the bound POPA molecule is directed toward the core of the TREK1 protein (Fig. 3a, d). This lipid tail encroaches on a region of TREK1 that houses the binding site for the TREK1 activators ML335 and ML402^32,34^ and contains the W275 and G137 residues, well-characterized positions that potentiate K2P currents when perturbed by mutation^9,10^. These TREK1 activating drugs and mutations share a common mechanism of action, exerting direct stabilizing effects on the nearby TREK1 selectivity filter gate to enhance channel activity^5,9,10,36^. Our structures suggest that a lipid bound at this upper site may similarly “bypass” the influence of the TM4 helix position to directly modulate the TREK1 selectivity filter.

We find that the presence of a lipid tail in the upper binding site forces an inward reorientation of the W275 sidechain toward the core of the protein, moving it closer to the G137 residue (Fig. 4d, e and Supplementary Fig. 4a–c). This previously unobserved inward movement of the W275 residue is a direct result of the presence of lipid in this binding pocket and is not simply reflective of TM4 helix positioning, as the rotameric orientation of the W275 sidechain in the POPA structure is distinct from the positioning of W275 in all our other TREK1 structures. In our POPE bound TM4 “down” structure and in both subunits of the apo TREK1 asymmetric TM4 “up/down” structure, W275 is positioned outward and is superimposable on the positioning of W275 in prior crystallographic TREK1 structures^32^ (Supplementary Fig. 4). The result of a bound lipid forcing W275 to shift to the inward-facing orientation is that W275 is then located in a position that overlaps with the dichlorophenyl group of a bound ML335 molecule or the isoleucine sidechain of the TRAAK G126I mutant, a structurally characterized gain of function mutation in TRAAK^5^ at a position equivalent to the TREK1 G137 (Supplementary Fig. 4). In all cases, a hydrophobic moiety (the inwardly oriented W275 sidechain, the isoleucine of the G137I mutant, or the ML335 molecule) becomes lodged at a site behind the selectivity filter pore loops (Fig. 4e and Supplementary Fig. 4), poised to directly influence the TREK1 selectivity filter. Mutations to either W275 or G137 are known to increase the open probability of the TREK1 channel and we suggest that these previously well-characterized mutations^5,9,10,13,36^ or the pharmacology that targets this modulatory pocket^32,34^ may in fact be recapitulating the actions of endogenous K2P lipid modulators, a pattern of overlap between lipids and pharmacology found in other ion channel families^37^.

### POPA closes the lateral fenestration and locks TM4 in the “up” state

The POPA density located at the lower binding site is positioned between the TM4 and TM1 helices, such that this binding site is only constituted when TM4 approaches TM1 in the “up” state and is broken in the TM4 “down” state (Figs. 4c, 6). An array of basic residues from both TM4 and TM1 surround the negatively charged headgroup of the PA lipid (Fig. 4b, c), suggesting a clear electrostatic basis for the binding of PA and other anionic lipids at this site. This POPA binding site is reminiscent of the “classical” PIP_2_ modulatory binding site found in numerous other potassium channels^38–41^ and prior studies of lipid binding to TREK1^21^ have shown that PIP_2_ and PA lipids compete for a shared TREK1 site. In fitting with our data, recent studies of TREK1 have modeled a PIP_2_ molecule in this region^34,42^, overlapping with the identified POPA molecule in our structure. Mutations to the polybasic stretch of residues on TM4 between R297 and K304 have already been shown to alter TREK1 sensitivity to PIP_2_^21,43^, providing functional corroboration for the role of these positively charged residues in constituting this anionic lipid binding site. The S300 residue, a protein kinase C phosphorylation target in the proximal C-terminus of TREK1^44^, lies directly in the center of the lower POPA binding site, 4.8 Å away from the well-resolved POPA headgroup (Fig. 4c). In this location, phosphorylation of S300 would be predicted to create both electrostatic and steric clashes with the headgroup of a bound anionic lipid and would disfavor the anionic lipid bound TM4 “up” state. Consistent with this structural observation, S300 phosphorylation or an S300D phospho-mimicking mutation are known to decrease TREK1 basal activity and responsiveness to activating stimuli^44,45^.

### Pore block by the zwitterionic lipid POPE

**Fig. 5:**
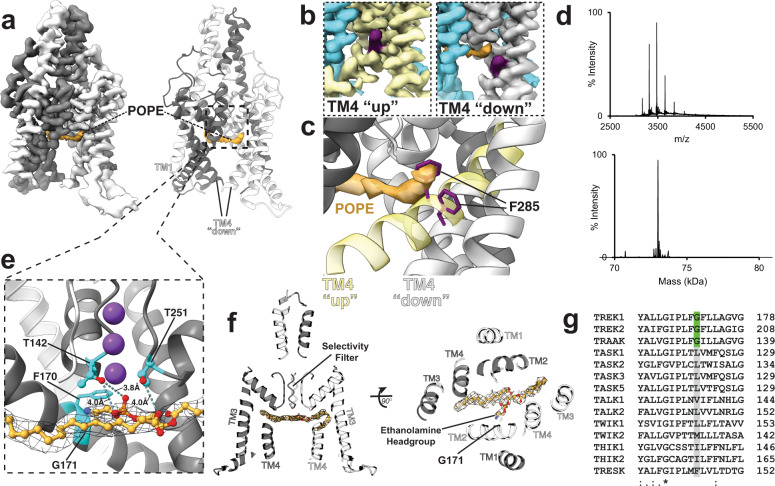
Headfirst pore block by POPE lipids. Cryo-EM map and matching structural model of TREK1 in DDM supplemented with POPE (**a**), highlighting the location of a single POPE lipid (orange) bound within the pore. The lipid density shown is derived from the sharpened map of the final refinement before C2 symmetry was applied, visualized at a contour threshold of 0.0402. A zoomed-in representation of the POPE binding site (**b**), highlighting the movement of the TM4 F285 residue (purple) when the POPE acyl chain tail occupies the pore site to shift TM4 from the “up” conformation (yellow, left panel) to the TM4 “down” conformation (white, right panel). Overlaying these two structural models (**c**) shows how the POPE molecule and the TM4 F285 residue would overlap if TM4 remained in the “up” conformation in the presence of POPE. **d** Native mass spectrum of TREK1 dimer with POPE (upper panel), and deconvoluted spectrum (lower panel), showing no POPE bound to TREK1. **e** A zoomed-in view of the interactions between the POPE headgroup and the TREK1 pore. **f** A structural model of TREK1 with the cryo-EM density of the POPE lipid shown in mesh, highlighting the position of the two lipid tails and the ethanolamine headgroup of the lipid. Each of the lipid tails are directed toward opposing fenestrations, and the position of TREK1 G171 is noted. **g** Sequence alignment of the TM2 helix from all human K2P channels, highlighting the alignment at the TREK1 G171 position.

While the POPA bound TREK1 structure is in a conductive TM4 “up” conformation, the addition of POPE lipids to TREK1 induced a pore-blocked TM4 “down” state, with unambiguous evidence for a POPE lipid located within the pore, directly below the selectivity filter (Fig. 5). The 3.2 Å local resolution at the center of the TREK1 POPE cryo-EM map (Supplementary Fig. 9) allowed us to model the entire POPE lipid within the TREK1 pore (Supplementary Fig. 5a, d). Mirroring our observation for the pore-bound DDM molecule in the TREK1 apo structure, the POPE lipid is oriented in an inverted pose, with the hydrophilic ethanolamine headgroup positioned centrally and each lipid tail splayed apart to occlude opposing open fenestrations (Fig. 5a, f). The presence of the POPE lipid forces both TM4 helices into the symmetric “down” state of the channel, occupying the position where the TM4 F285 sidechain is located when TM4 is in the “up” conformation (Fig. 5b, c). This rearrangement mirrors a movement of an equivalent phenylalanine in the KcsA potassium channel (KcsA F103) that occurs upon binding of quaternary ammonium blockers to a similarly located binding site below the KcsA pore^46^. Cryo-EM density for the ethanolamine headgroup of the POPE lipid is visible within the pore vestibule, occluding the ion permeation pathway. The observed pore blocking density in the POPE structure differs in shape from the DDM density observed within the pore of the apo TREK1 structure (Fig. 1c, d and Supplementary Fig. 5), though both pore blocking densities occupy an overlapping site below the selectivity filter. Modeling of a POPE molecule into this density reveals that the POPE headgroup is poised to interact with TREK1 residues that comprise the S4 potassium binding site of the selectivity filter (Fig. 5e). TREK1 T142 from selectivity filter pore loop 1 is positioned to hydrogen bond with the oxygen atoms of the POPE phosphate headgroup while TREK1 T252 from pore loop 2 can hydrogen bond with the sn2 oxygen of the POPE glycerol backbone. The POPE ethanolamine group points toward a TM2 glycine at position 171, a residue that is a conserved glycine only in the mechanosensitive subfamily of K2Ps (Fig. 5g, TREK1, TREK2, and TRAAK). The absence of a larger amino acid sidechain at this position appears to create sufficient space for the pore to accommodate the ethanolamine headgroup (Fig. 5e, f).

While most of the particles in the TREK1 POPE dataset are in the TM4 “down/down” state, this lipid condition exhibits more heterogeneity than the TREK1 apo or TREK1 POPA datasets, with ~30% of the TREK1 POPE particles in an asymmetric TM4 “up/down” conformation (Supplementary Fig. 8). A 3D reconstruction of these TM4 “up/down” particles yielded a 3.83 Å resolution map, structurally indistinguishable from the TREK1 apo TM4 “up/down” conformation and similarly featuring an asymmetrically positioned pore blocking density below the selectivity filter (Supplementary Fig. 5f, g). At the relatively lower resolution of this reconstruction, it is not possible to definitively determine the nature of this density. One possibility is that this less populated class is comprised of apo TREK1 channels that have not exchanged the pore-bound DDM for a POPE lipid. Alternatively, the asymmetric POPE structure could reflect a secondary location for the bound POPE molecule, with its headgroup located in the pore and acyl tails directed toward only one fenestration. We hypothesize that such a state can exist as an intermediate between the TM4 “up” conductive state of the channel and one that is blocked by a POPE lipid with its headgroup positioned below the pore and hydrocarbon tails splayed apart.

Despite the clear presence of a POPE lipid bound within the pore vestibule in the symmetric TM4 “down” structure, native MS analysis of TREK1 in the presence of POPE did not reveal evidence of POPE bound to DDM solubilized TREK1 (Fig. 5d). This discordance between the native MS data and our structural results suggests that the pore blocking lipid identified in our cryo-EM structure is only loosely bound within the identified pore site and may readily exit the pore under the ionization and activation conditions required for native MS of TREK1 in DDM. This result contrasts with a native MS study of TRAAK showing multiple bound POPE to TRAAK at similar POPE concentrations. This discrepancy may be due to the use of C10E5 detergent in the TRAAK study, which permits gentler ionization conditions^35^. We found that TREK1 was not stable when exchanged into C10E5 detergent. Nevertheless, given that anionic POPA lipids are readily observed to bind to the TREK1 protein in DDM by native MS (Fig. 3b, d), the absence of POPE binding under identical ionization/activation conditions suggests that the upper and lower anionic lipid binding sites identified in our TREK1 POPA structure (Fig. 3) are not targeted by the zwitterionic lipid POPE. This result implicates lipid headgroup chemistry as a key determinant of lipid selectivity at these anionic sites, an assertion supported by the absence of evidence for lipid densities at either the upper “classical” or lower “bypass” lipid binding sites in the TREK1 POPE cryo-EM structure.

## Discussion

Since the determination of the first K2P channel structures^20,47^, the presence of membrane-accessible fenestrations in the K2P channel architecture suggested that lipids might play a role in controlling K2P gating. While an initial conception of K2P lipid block involved lipid acyl chain tails extending from the bilayer into the channel pore to block conduction, MD simulations of TREK2 channels suggested that phospholipid tails were in fact too short to occlude the pore cavity in such a manner^48^. Our study elucidates an alternative mechanism for the phospholipid block of K2Ps. In both the TREK1 apo and TREK1 POPE cryo-EM structures, it is the hydrophilic portion of the amphipathic detergent or lipid molecule that occupies the channel pore, not the hydrocarbon tails. These findings suggest that membrane phospholipids may enter the TREK1 vestibule in an inverted, headfirst orientation to block the pore.

**Fig. 6:**
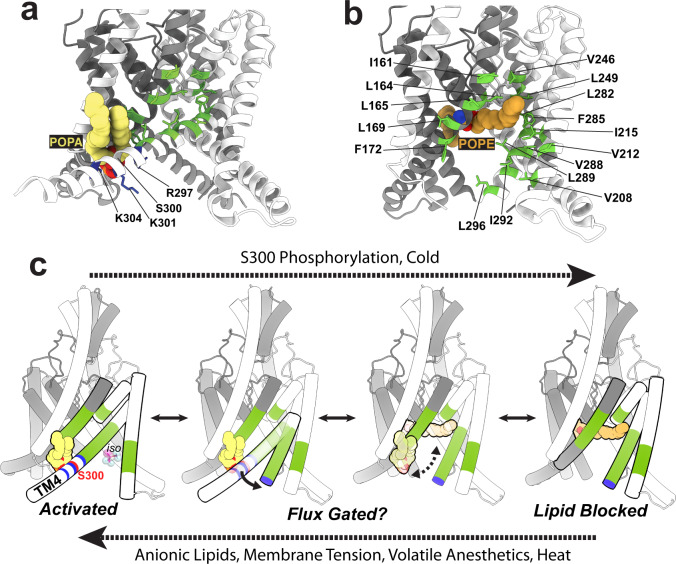
An integrated model of TREK1 lower pore gating. Comparison of the POPA and POPE bound TREK1 structures. In the POPA structure (**a**), the lipid pathway is closed, with no lipid occluding the ion-conducting pore and positively charged residues (blue) in the distal end of TM4 enveloping the anionic POPA headgroup. In the POPE structure (**b**), hydrophobic residues (green) on TM2, TM3, TM4, and pore helix 2 line the open lipid pathway. **c** A cartoon schematic describing the proposed integrated model of lower pore gating in TREK1 channels. In the leftmost panel, iso represents the binding site position of the volatile anesthetic isoflurane^53^.

For a phospholipid to enter the TREK1 pore in this inverted orientation, there needs to be an energetically favorable pathway for a lipid headgroup to reach the core of the protein within the membrane. In our structure of TREK1 in the TM4 “down” state, the open fenestration formed by the TM2, TM3, and TM4 helices creates such a pathway; a membrane-exposed groove lined by hydrophobic amino acids with direct access to the pore vestibule (Fig. 6b). This groove is closed in the TM4 “up” conformation of the channel, though a lipid bound at the lower anionic binding site is poised at the mouth of the closed pathway (Fig. 6a). When TM4 moves to the “down” position, the lipid pathway opens and becomes exposed to the inner leaflet of the bilayer. Lipid acyl chains are free to enter the open groove and become enveloped by the hydrophobic residues lining the pathway, while the hydrophilic headgroups can position themselves within the hydrated channel pore. We speculate that in a biological membrane, phospholipids may fill the region between the pore-bound POPE lipid site and the lower POPA site when the lipid pathway is open, with our cryo-EM structures defining the structurally stabilized outer delimiting positions for phospholipid binding to TREK1.

While further experimental and molecular dynamic work will be required to explore the energetics of such a proposed lipid path within the TREK1 channel, recognition of the possibility of this pore-accessible lipid pathway in TREK1 provides a framework for an overarching mechanism of lower pore gating in mechanosensitive K2Ps (Fig. 6c). In this model, TREK1 activating stimuli including anionic lipids, membrane tension, and volatile anesthetics all converge to promote the TM4 “up” conformational state and unblock the TREK1 pore. For the case of modulatory anionic phospholipids like PA, the net negative charge of an anionic lipid headgroup would be expected to disfavor entry into the vestibule of the cation-selective TREK1 pore. Local increases in bilayer concentrations of PA (as can naturally occur through activation of TREK1 bound phospholipase D2^23^) would thus reduce lipid occupancy within the pore, promote the conductive TM4 “up” conformation, and constitute the lower anionic lipid binding site to further stabilize TM4 in the active “up” state. A similar argument applies to membrane tension, which has also been predicted to favor the TM4 “up” state^12,49^. The peak of the trans-bilayer pressure profile induced by a mechanical stimulus is believed to arise at the water lipid interface^50^, located at the mouth of the lipid-accessible groove. This suggests that the application of membrane tension will remove lipids from the membrane-accessible path, allowing TM4 to shift to a conductive TM4 “up” conformation. Such a mechanism would explain the convergent influences of positive allosteric anionic lipids and mechanosensitivity^24,25^ and mirrors a force-from-lipid-based mechanism of mechano-gating demonstrated in the prokaryotic ion channel MscS, where membrane tension first removes lipids from key regions of the MscS structure to subsequently unlock conformational changes that open the MscS pore^51,52^. Volatile anesthetics (VAs) also appear to favor the TM4 “up” conformation of TREK1, as the VA binding site identified in TREK1^53^ becomes incorporated into the lipid-accessible pathway when TM4 is “down” and is only accessible to a VA when TM4 moves into the “up” conformation (Fig. 6c). This model of TREK1 lower gating also incorporates the inhibitory influences of cold temperature and S300 phosphorylation, both of which appear to share a convergent effect on TREK1 activity^44,45^. Temperature sensing is not believed to be an intrinsic property of TREK1 but is rather tied to modulation of the phosphorylation state of the TREK1 S333 and S300 residues^44,54^. With S300 located at the center of the lower anionic lipid binding site (Fig. 4c), phosphorylation of the S300 residue would be expected to disrupt anionic lipid binding, favor the pore-blocked TM4 “down” conformation, and inhibit channel activity.

While our study identifies important lipid binding sites in the TREK1 protein, the composition and distribution of lipids in the lipid-detergent mixed micelles utilized in our work deviate from the composition of native mammalian membranes. Whereas the zwitterionic lipid POPE comprises ~30% of the lipid in the mammalian membrane^55^, we are limited to ~10 mol% POPE in our structural work, a concession necessitated by the solubility limit of POPE in the DDM detergent. At this 10% POPE molar fraction, we observed both functional and structural effects of POPE on TREK1 but were unable to observe binding of the POPE lipid to the TREK1 protein by native MS. We speculate that for a presumed low-affinity POPE binding site that is exposed to a very high molar fraction of zwitterionic lipid in biological membranes, we may simply not be at a high enough concentration of POPE in our micelles to observe TREK1/POPE binding by native MS. In contrast to POPE, the anionic lipid POPA is a low-abundance lipid (~1%) in the mammalian membrane, generally segregated to the inner leaflet of the bilayer. The presence of an anionic phospholipid binding site at the extracellular face of TREK1 was therefore somewhat surprising to us, given the low abundance of anionic lipids in this leaflet of biological membranes. While local activation of PLD2^23^ may temporally and locally boost the concentration of PA lipids near the TREK1 protein to approximate the concentrations of POPA used in our structural studies, it is also possible that low-abundance anionic phospholipids are not the only substrate that binds to the selectivity filter adjacent lipid binding site. Both lysophospholipid^56^ and eicosanoid^57^ signaling lipids have been found to act at the external face of the TREK1 channel and it is possible that the extracellular POPA binding site identified in our study also serves as a target for these important regulatory signaling lipids. While our structures demonstrate the impact of the insertion of a lipid tail into the pocket behind the selectivity filter, the local resolution is insufficient to define the molecular interactions between the POPA headgroup and the extracellular face of the TREK1 protein. A loop region between pore helix 2 and the upper TM4 lies directly above the POPA lipid headgroup and we hypothesize that this region of the K2P structure imparts lipid selectivity at the upper binding site. Supporting this notion is the observation that this region of the TREK1 protein is responsible for the divergent sensitivities of TREK1 and TREK2 channels to external pH^58^ and the eicosanoid lipid prostaglandin F2α^57^ and is also a key determinant of ML335 and ML402 affinity^32^.

It is important to note that there is additional complexity to K2P gating that we cannot directly address from our results in this study. While our findings reinforce an emerging consensus that the TM4 “up” conformation of TREK1 is the stimulus-activated state of the channel, there is clear evidence for a conductive TM4 “down” state in many K2Ps^12,13,18,19^. Functional studies of TREK1 have shown that activating stimuli convert the channel from a voltage-dependent pattern of behavior to an open rectifying leak current^19,59^, suggesting that the conductive TM4 “down” state is the structural correlate of this voltage-gated functional behavior. As K2P channels do not have a canonical S1-S4 voltage sensor, voltage dependence is believed to arise within the pore, with the direction of ion flow implicated as a key to this behavior, termed “flux gating”^19^. We postulate that TREK1 pore occupancy by lipids could be influenced by the direction of ion flow across the K2P pore, linking lipid block to the voltage-dependent behavior of a conductive TM4 “down” state of TREK1. However, without a structural correlate of this conductive TM4 “down” state in TREK1, this assertion remains an untested hypothesis. A recent study of the mechanosensitive K2P TRAAK did determine a conductive TRAAK TM4 “down” structure by introducing a point mutation within the TRAAK pore^12^. The site of this point mutation in TREK1 is the TM2 G171, the residue that directly opposes the ethanolamine headgroup of the pore-bound POPE molecule in our TM4 “down” structure (Fig. 5e, f). Mutations at this position cause potentiation of channel function in all K2P subfamilies^60^, though we note that this pore lining TM2 residue is a conserved glycine only in mechanosensitive K2Ps (TREK1, TREK2, and TRAAK) and is a bulky hydrophobic residue in all other K2P channels (Fig. 5g). While this suggests that the pore of mechanosensitive K2Ps may be uniquely conducive to accommodating a lipid headgroup at a site directly below the selectivity filter, the question of whether lipid block is an important gating modulator in other K2Ps subfamilies remains intriguing but as of yet unanswered.

## Methods

### Purification of TREK1 protein

For structural studies of TREK1, we utilized a well-characterized and biochemically tractable C-terminally truncated zebrafish ortholog of the TREK1 channel (drTREK1), a construct previously shown to retain sensitivity to many important TREK1 modulatory signals, including mechanical stretch, heat, arachidonic acid, volatile anesthetics and the TREK1 activator BL1249^22,53^. The TREK1 ortholog is 89% sequence similar and 78% sequence identical to a matched region of the human TREK1 ortholog (Supplementary Fig. 1), with much of the sequence divergence clustered in the N-terminal and helical CAP domain regions of the protein sequence.

drTREK1 protein was expressed in *Pichia pastoris* (Thermo Fisher, SMD1168H, Catalog # C18400) using a previously described pPICZ vector bearing residues 1–322 of the *D. rerio* TREK1 gene (TREK1, K2P2.1, UniProt X1WC65) with mutations introduced to eliminate N-linked glycosylation sites (N95Q, N122Q), followed by a PreScission protease-cleavage site (LEVLFQ/GP) and C-terminal GFP and His_10_ tags^22^. The expression plasmid was linearized with the PmeI restriction enzyme and subsequently transformed into *P. pastoris* by electroporation. Screening for successful recombinant integration was performed by plating transformants on yeast extract peptone dextrose sorbitol (YPDS) plates containing increasing concentrations of zeocin from 0.5 to 3 mg/ml and incubating for 3–5 days at 30 °C until colonies appeared.

Yeast transformants were grown in a buffered minimal medium (2 × YNB, 1% glycerol, 0.4 mg L^−1^ biotin, 100 mM potassium phosphate [pH 6.0]) for 2 days at 30 °C in a shaker at 225 rpm. Cells were pelleted by centrifugation (4000×*g* at 20 °C, 5 min) and resuspended in methanol minimal medium (2 × YNB, 0.5% methanol, 0.4 mg L^−1^ biotin, 100 mM potassium phosphate [pH 6.0]) to induce protein expression. Cells were then shaken for 2 additional days at 22 °C in a shaker at 225 rpm, with additional methanol added (final concentration 0.5% [v/v]) to the culture after 24 h of protein expression. After 48 h of protein expression, cells were pelleted by centrifugation (6000×*g* at 4 °C for 10 min), flash frozen in liquid nitrogen, and subjected to three rounds of cryo-milling (Retsch model MM301) in liquid N_2_ for 3 min at 25 Hz to disrupt yeast cell walls and membranes. Frozen yeast cell powder was stored at −80 °C until use.

To purify TREK1, cell powder was added to breaking buffer (150 mM KCl, 50 mM Tris pH 8.0, 1 mM phenylmethylsulfonyl fluoride, 0.1 mg/ml DNase 1, and 1 tablet/50 ml of EDTA-free complete inhibitor cocktail [Roche]) at a ratio of 1 g cell pellet/2 ml lysis buffer. Solubilized cell powder was centrifuged at 4000×*g* at 4 °C for 5 min to pellet large debris and the supernatant was then centrifuged at 100,000×*g* at 4 °C for 1.5 h to pellet cell membranes. The pellet was resuspended in 50 ml breaking buffer containing 60 mM *n*-Dodecyl-B-d-Maltoside (DDM) and incubated for 3 h with gentle stirring to solubilize the membranes, followed by centrifugation at 35,000×*g* for 50 min. Talon cobalt resin (Takara Bio USA) was added to the supernatant at a ratio of 1 ml of resin per 10 g of cell powder and incubated in an orbital rotor overnight at 4 °C. Resin was then collected on a column and washed with 10-column volumes of Buffer A (150 mM KCl, 50 mM Tris pH 8.5, 6 mM DDM, 30 mM imidazole) and bound protein was subsequently eluted from the resin by washing with Buffer B (150 mM KCl, 50 mM Tris pH 8.5, 6 mM DDM, and 300 mM imidazole). PreScission protease (∼1:25 wt:wt) was added to the eluate and the cleavage reaction was allowed to proceed overnight at 4 °C under gentle rocking. Cleaved TREK1 protein was concentrated in 50 kDa molecular weight cutoff (MWCO) Amicon Ultra Centrifugal Filters (Millipore) and applied to a Superdex200 10/300 gel filtration column (GE Healthcare) equilibrated in size exclusion chromatography (SEC) buffer (150 mM KCl, 20 mM Tris pH 8.0, 1 mM DDM). Purified TREK1 protein was concentrated (50 kDa MWCO) to 3 mg/ml and analyzed for purity by SDS-PAGE [12% (wt/vol) gels; Bio-Rad] followed by staining with Coomassie blue. All protein purification steps were carried out at 4 °C.

For the preparation of DDM/lipid-mixed micelle samples, purified TREK1 protein was first subjected to two additional runs over a Superdex200 10/300 gel filtration column in size exclusion chromatography buffer containing ~2x CMC concentration of DDM detergent (150 mM KCl, 20 mM Tris pH 8.0, 0.25 mM DDM) and then an additional third gel filtration run in buffer containing dissolved 16:0–18:1 phosphatidic acid (POPA) or 16:0–18:1 phosphatidylethanolamine (POPE) (Avanti). POPA was dissolved at a concentration of 0.1 mg/ml, whereas POPE was dissolved at 0.025 mg/ml, the empirically determined solubility limit of POPE in 0.25 mM DDM buffer.

### ACMA fluorescence quenching assay of TREK1 function

To prepare TREK1 proteoliposomes in varied lipid compositions, chloroform solubilized 18:1–18:1 phosphatidylcholine (DOPC), POPA, or POPE lipids were combined in borosilicate glass vials to a final lipid concentration of 5 mg total lipid per reconstitution. Lipids were dried under nitrogen, washed once with pentane to remove residual chloroform, and redried into a thin lipid film under nitrogen, followed by overnight incubation under vacuum in a vacuum desiccator to fully dry the lipid mixtures. Lipids were then solubilized in HighK buffer (150 mM KCl, 20 mM HEPES, pH 7.4) supplemented with 8 mM CHAPS and sonicated until the solution was visually clear. The solubilized lipids were mixed with purified TREK1 protein (0.5 μg/mg lipid, unless otherwise indicated) and allowed to incubate at room temperature for 20 min, followed by the addition of 200 mg SM-2 biobeads (Bio-Rad) to remove the detergent and form proteoliposomes. Samples were rotated in the presence of biobeads for 2 h at room temperature and the formed proteoliposomes were then extruded through a 0.1 µm filter (Whatman) using a mini-extruder (Avanti Polar lipids), to produce uniform ~100 nm liposomes ready for ACMA studies. Proteoliposomes were made fresh for each experiment and used within 24 h of preparation.

For 9-Amino-6-Chloro-2-Methoxyacridine (ACMA) fluorescence quenching assays, our protocols mirror those of prior studies utilizing this assay for the study of K2P function^21,35,61^. About 50 μl of freshly prepared TREK1 proteoliposomes were mixed with 2 ml of HighNa buffer (150 mM NaCl, 20 mM HEPES, pH 7.4) containing 2 μM ACMA and transferred to quartz cuvettes (Hellma) in a PTI quantamaster fluorimeter. Sixty seconds of baseline recording was captured (measurements taken every 0.2 s), with excitation at 410 ± 9 nm and emission measured at 490 ± 15 nm. ACMA quenching was initiated by the addition of the proton ionophore carbonyl cyanide 3-chlorophenylhydrazone (CCCP) to a final concentration of 1 μM. After quenching reactions plateaued, the potassium ionophore valinomycin was added to the reactions (final concentration 18 nM) to collapse the potassium gradient across the proteoliposome membrane and complete ACMA quenching. All traces were normalized to their individual baseline and post-valinomycin treated values (F_start_ – F)/(F_start_ - F_valin_), with F_start_ defined as the final value of the first 60 s of recording prior to the addition of CCCP and F_valin_ as the final time point in the recording once valinomycin treatment was completed.

### Native mass spectrometry and analysis of lipid extracts

Purified TREK1 in DDM was analyzed by native MS on a Q-Exactive EMR as previously described^62^. About 50 μl of TREK1 at 1 mg/ml in 150 mM KCl, 20 mM Tris pH 8.0, 0.25 mM DDM was buffer exchanged into 200 mM ammonium acetate pH 8 and 0.25 mM DDM using Biospin gel filtration columns (Bio-Rad). For the TREK1 samples containing POPA or POPE, buffer exchange was performed using 200 mM ammonium acetate pH 8 and 0.25 mM DDM, supplemented with either 0.1 mg/ml POPA or 0.025 mg/ml POPE. This maintained the same phospholipid concentration that was used for cryo-EM. About 3 μl of this sample was directly loaded into a borosilicate capillary emitter (Thermo Scientific, ES380) and native MS spectra were obtained by static nanospray on a Thermo Q-Exactive EMR mass spectrometer. For the no-lipid, POPA and POPE samples, the data were collected using an electrospray voltage of 1.5 kV, capillary temperature of 200 °C, resolution of 8750, trap and transfer voltages of 200 V (CID) and 150 V (CE), respectively, and HCD pressure as measured by the UHV pressure gauge set to 8.9 e-10 mbar. Ion transfer optics were set at 8, 7, 6, and 4 V for injection flatapole, inter-flatapole lens, bent flatapole, and transfer multiple, respectively. The spectra in Fig. 3c and Supplementary Fig. 2f, g was collected by increasing CID to 200 V and CE to 200 V and decreasing the HCD pressure to a UHV pressure of 4.0 e-10 mbar. Greater activation by further decreasing the HCD pressure led to a loss of the TREK1 dimer signal. The native MS spectra were deconvoluted using UniDec Version 3.2.0. The spectra were processed from m/z 1,500 to 15,000, curved background subtraction was applied, and “0.0” was indicated for binning of data points. Deconvolution was performed with a charge range of 1 to 50, a mass range of 5,000 to 100,000 Da, and a resolution of 1.0 Da for the zero-charge spectrum^63^.

Analysis of lipid extracts from purified TREK1 were performed as previously described^62^. We performed a Bligh-Dyer extraction^64^ using 100 μg of purified TREK1 with and without ~1 μg of POPA (1:1 ratio of TREK1 dimer: POPA). After the extraction, the organic phase was removed and dried using nitrogen gas. The sample was then reconstituted in 100 μl of 1:1 methanol:chloroform and 0.5% ammonium hydroxide, and analyzed by direct injection on a Thermo Elite mass spectrometer using a Max Ion API source with a HESI-II probe. MS1 spectra were acquired at 120,000 resolution from 250–2000 m/z in negative ion mode with a flow rate of 5 μl/min, electrospray voltage of 4 kV, and capillary temperature of 300 °C. MS2 spectra were acquired with CID using an isolation window of 1 Da, relative collision energy of 30%, activation q value of 0.25 and activation time of 10 ms. For analysis by positive ion mode, the samples were prepared with and without ~1 μg of POPC and POPE (1:1 ratio of TREK1 dimer: POPC or POPE), reconstituted in 1:1 methanol:chloroform with 0.1% formic acid after the Bligh-Dyer extraction, and injected with an electrospray voltage of 4 kV and the same parameters as above. MS2 spectra were collected for all peaks ranging from 600–850 m/z. The MS1 and MS2 spectra were manually analyzed using tools on www.lipidmaps.org.

To search for ergosterol in the purified TREK1 sample, we performed GC/MS analysis using TMS derivatization of the lipid extract. A Bligh-Dyer extraction was performed on 100 μg of purified TREK1 along with 2.7 nmol of cholesterol as an internal standard (1:1 TREK1 monomer: cholesterol). The organic phase was blown dry and then derivatized with 65 μl of the derivatization reagent (1:0.4:2.6 *N*-methyl-*N*-trimethylsilyltrifluoroacetamide with 1% trimethylchlorosilane:pyridine:acetonitrile) and heated at 65 °C for 1 h. Electron ionization GC/MS was performed on an Agilent 7890 A GC/5975 C MS, using a 25-m Agilent J & W capillary column (DB-1; inner diameter, 0.25 mm; film thickness, 0.1 m). The GC inlet and transfer line temperatures were set to 250 and 280 °C, respectively; and 2 μl of the derivatized sample was injected in a splitless mode. The temperature program started at 80 °C for 2 min, increased at 50 °C/min to 268 °C, held for 1 min, increased by 1 °C/min to 275 °C, ramped to 282 °C at 5 °C/min, and then reached to 300 °C at 20 °C/min and was held for 8 min. The mass spectra were acquired both in full scan (scan range 60–650 Da) and selected ion monitoring (SIM) modes. SIM ions at m/z 329, 368, and 458 for cholesterol and at m/a 468, 363, and 337 for ergosterol were monitored.

### Cryo-EM grid preparation and data collection

TREK1 protein samples were frozen on R1.2/1.3 UltraAufoil 300 mesh grids (Quantifoil), prepared for sample application by glow discharge for 80 s at +25 mA using a Pelco easiGlow glow discharge system (Ted Pella). A volume of 4 μL of 3 mg/ml purified TREK1 protein sample was applied to each grid and freezing was performed using a Vitrobot Mark IV (FEI). Samples were equilibrated on the glow discharged grids in the Vitrobot chamber at 21 °C and 100% humidity for 20 s and then blotted for 1.5 s with +4 blot force before being plunge frozen in liquid ethane.

For the TREK1 apo dataset, movies were acquired at 64,000x nominal magnification on a Titan Krios microscope (FEI) operated at 300 kV, equipped with a K3 direct electron detector (Gatan) and a GIF quantum energy filter (20 eV) (Gatan). Data were collected using Leginon software^65^ at a dose rate of 25.92 e^-^/Å^2^/s with a total exposure of 2.00 s, for an accumulated dose of 51.83 e^-^/Å^2^. Intermediate frames were recorded every 0.04 s for a total of 50 frames per micrograph, at a nominal defocus range of 0.8–4.0 μm. A calibrated pixel size of 0.5413 Å was used for processing.

An alternative Titan Krios microscope was used for the collection of TREK1 datasets in lipid-mixed micelles (POPA or POPE). In both cases, the microscope was operated at 300 kV and images were acquired at 105,000x nominal magnification using a K3 direct electron detector (Gatan). Data were collected using Leginon software^65^. For the POPA dataset, movies were collected at a dose rate of 23.45 e^-^/Å^2^/s with a total exposure of 2.70 s, for an accumulated dose of 63.32 e^-^/Å^2^. Intermediate frames were recorded every 0.06 s for a total of 45 frames per micrograph, at a nominal defocus range of 1.3–1.8 μm. For the POPE dataset, movies were collected at a dose rate of 22.43 e^-^/Å^2^/s, with a total exposure of 2.40 s, for an accumulated dose of 53.83 e^-^/Å^2^. Intermediate frames were recorded every 0.05 s for a total of 48 frames per micrograph, at a nominal defocus range of 1.3–2.0 μm. A calibrated pixel size of 0.426 Å was used for processing both the POPA and POPE datasets.

### Cryo-EM data processing and model building

All three datasets were processed in RELION 3.1^66^, using the same general workflow. Pertinent details of all datasets are presented in Supplementary Table 1. Dose-fractionated images were either 2× Fourier binned (TREK1 Apo dataset) or 3x Fourier binned (TREK1 POPA and POPE datasets), gain normalized, and dose-weighted using RELION’s implementation of Motion-Cor2^67^, with micrographs processed in 10 × 10 patches. Contrast transfer function (CTF) and defocus estimation was performed using CTFFIND4^68^. Single particles were initially autopicked in RELION by a Laplacian of Gaussian (LOG) approach and subjected to reference-free 2D classification, generating references for use in a second round of 2D reference-based autopicking. Ab-initio models were generated from the subset of particles in the LOG-picked 2D reference classes and the best ab-initio model was used as the input for the 3D classification of the final 2D reference-picked particle stack. All 3D classes with evidence of transmembrane domain density were pooled and subjected to 3D refinement, with the best 3D class used as an initial model (low pass filtered to 20 Å). Maps were improved through Iterative rounds of particle polishing, beam tilt estimation, anisotropic magnification estimation, and per-particle CTF estimation, followed by 3D refinement, until convergence. Any remaining low-quality particles were then removed from the dataset by a combination of 3D classification without alignment and additional rounds of 2D classification, selecting classes with the strongest transmembrane domain signals relative to the micelle density. A final refinement utilizing SIDESPLITTER^69^ to mitigate local overfitting (most prevalent in the TM2/TM3 loop) improved both final resolution values and map quality. Reported resolution at an FSC cut-off of 0.143 was determined using RELION postprocessing, performed with a volume mask designed to exclude the detergent micelle. Local resolution estimates were calculated in Relion.

For model building and data representation, unmasked and unfiltered half-map reconstructions of the final refinements from each dataset were sharpened using DeepEMhancer software^70^. The TREK1 Apo and TREK1 POPA models were built using the final maps shown in Supplementary Figs. 6,7, respectively. For the TREK1 POPE dataset, the model was built using two distinct cryo-EM maps, as denoted in Supplementary Fig. 8. The final C2 symmetrized reconstruction was used to model the TREK1 protein chains, while the final C1 reconstruction was used to build the centrally positioned POPE lipid. C2 symmetry improved the resolution of the TREK1 POPE reconstruction and aided in a model building but distorted the centrally positioned POPE lipid, necessitating the use of the final C1 reconstruction (without any applied symmetry) to model the POPE lipid.

A previously published crystal structure of mouse TREK1 in the TM4 “up” state (PDB: 6CQ6)^32^ was used as a starting point for model building and was docked into the sharpened apo TREK1 cryo-EM density map using UCSF chimera^71^. This model was manually adjusted in COOT^72^ to fit into the cryo-EM density map and the primary sequence of the mouse TREK1 model was mutated as appropriate to produce a zebrafish TREK1 starting model. Following manual building, global real space refinement with stereochemistry restraints was performed using Phenix^73^. Sidechain outliers present after real space refinement were individually inspected and corrected in COOT^72^. After the TREK1 apo model had been built, it was used as a starting point for building the lipid-bound structures of TREK1 in alternative conformations, using an equivalent approach.

### Reporting summary

Further information on research design is available in the Nature Portfolio Reporting Summary linked to this article.

## Supplementary information

Supplementary Information

Reporting Summary

## Data Availability

Data supporting the findings of this manuscript are available from the corresponding author upon request. Cryo-EM maps of TREK1 have been deposited in the Electron Microscopy Data Bank (EMDB) under accession codes: EMD-27386 (TREK1 in DDM detergent); EMD-27387 (TREK1 in DDM/POPA mixed micelles); EMD-27388 (TREK1 in DDM/POPE mixed micelles). Atomic coordinates for all structures have been deposited in the Protein Data Bank (PDB) with accession codes 8DE7 (TREK1 in DDM detergent); 8DE8 (TREK1 in DDM/POPA mixed micelles); 8DE9 (TREK1 in DDM/POPE mixed micelles). A crystallographic-derived model of mouse apo TREK1 WT (6CQ6) was used for initial model building. The source data for all ACMA fluorescence quenching results is provided as a source data file. Source data are provided with this paper.

## Source data

Source Data

## References

[1] Plant LD (2012). A role for K2P channels in the operation of somatosensory nociceptors. Front. Mol. Neurosci..

[2] Honore E (2007). The neuronal background K2P channels: focus on TREK1. Nat. Rev. Neurosci..

[3] Heurteaux C (2004). TREK-1, a K+ channel involved in neuroprotection and general anesthesia. EMBO J..

[4] Enyedi P, Czirjak G (2010). Molecular background of leak K+ currents: two-pore domain potassium channels. Physiol. Rev..

[5] Lolicato M, Riegelhaupt PM, Arrigoni C, Clark KA, Minor DL (2014). Transmembrane helix straightening and buckling underlies activation of mechanosensitive and thermosensitive K(2P) channels. Neuron.

[6] Dong YY (2015). K2P channel gating mechanisms revealed by structures of TREK-2 and a complex with Prozac. Science.

[7] Brohawn SG, Campbell EB, MacKinnon R (2014). Physical mechanism for gating and mechanosensitivity of the human TRAAK K+ channel. Nature.

[8] Zhuo RG (2016). Allosteric coupling between proximal C-terminus and selectivity filter is facilitated by the movement of transmembrane segment 4 in TREK-2 channel. Sci. Rep..

[9] Bagriantsev SN, Peyronnet R, Clark KA, Honore E, Minor DL (2011). Multiple modalities converge on a common gate to control K2P channel function. EMBO J..

[10] Bagriantsev SN, Clark KA, Minor DL (2012). Metabolic and thermal stimuli control K(2P)2.1 (TREK-1) through modular sensory and gating domains. EMBO J..

[11] Piechotta, P. L. et al. The pore structure and gating mechanism of K2P channels. *EMBO J*. **30**, 3607–3619 (2011). 10.1038/emboj.2011.268 PMC3181484 21822218

[12] Rietmeijer, R. A., Sorum, B., Li, B., Brohawn, S.G. Physical basis for distinct basal and mechanically gated activity of the human K + channel TRAAK. *Neuron* **109**, 2902–2913 (2021). 10.1016/j.neuron.2021.07.009 PMC8448962 34390650

[13] McClenaghan C (2016). Polymodal activation of the TREK-2 K2P channel produces structurally distinct open states. J. Gen. Physiol..

[14] Zilberberg N, Ilan N, Goldstein SA (2001). KCNKO: opening and closing the 2-P-domain potassium leak channel entails “C-type” gating of the outer pore. Neuron.

[15] Cuello, L. et al. Structural basis for the coupling between activation and inactivation gates in K(+) channels. *Nature* **466**, 272–275 (2010). 10.1038/nature09136 PMC3033755 20613845

[16] Kopec, W., Rothberg, B. S. & de Groot, B. L. Molecular mechanism of a potassium channel gating through activation gate-selectivity filter coupling. *Nat. Commun.* **10**, 5366 (2019). 10.1038/s41467-019-13227-w PMC6879586 31772184

[17] Li, J., Ostmeyer, J., Cuello, L.G., Perozo, E. & Roux, B. Rapid constriction of the selectivity filter underlies C-type inactivation in the KcsA potassium channel. *J. Gen. Physiol.* **150**, 1408–1420 (2018). 10.1085/jgp.201812082 PMC6168234 30072373

[18] Proks, P. et al. Norfluoxetine inhibits TREK-2 K2P channels by multiple mechanisms including state-independent effects on the selectivity filter gate. *J. Gen. Physiol.* **153**, e202012812 (2021). 10.1085/jgp.202012812 PMC8155809 34032848

[19] Schewe M (2016). A non-canonical voltage-sensing mechanism controls gating in K2P K(+) channels. Cell.

[20] Miller, A. N. & Long, S. B. Crystal structure of the human two-pore domain potassium channel K2P1. *Science* **335**, 432–436 (2012). 10.1126/science.1213274 22282804

[21] Cabanos, C., Wang, M., Han, X. & Hansen, S. A. Soluble fluorescent binding assay reveals PIP 2 antagonism of TREK-1 channels. *Cell Rep.* **20**, 1287–1294 (2017). 10.1016/j.celrep.2017.07.034 PMC5586213 28793254

[22] Brohawn SG, Su Z, MacKinnon R (2014). Mechanosensitivity is mediated directly by the lipid membrane in TRAAK and TREK1 K+ channels. Proc. Natl Acad. Sci. USA.

[23] Comoglio Y (2014). Phospholipase D2 specifically regulates TREK potassium channels via direct interaction and local production of phosphatidic acid. Proc. Natl Acad. Sci. USA.

[24] Patel AJ (1998). A mammalian two pore domain mechano-gated S-like K+ channel. EMBO J..

[25] Honore E, Maingret F, Lazdunski M, Patel AJ (2002). An intracellular proton sensor commands lipid- and mechano-gating of the K(+) channel TREK-1. EMBO J..

[26] Kim, Y., Gnatenco, C., Bang, H. & Kim, D. Localization of TREK-2 K+ channel domains that regulate channel kinetics and sensitivity to pressure, fatty acids and pHi. *Pflugers Archiv.* **442**, 952–960 (2001). 10.1007/s004240100626 11680629

[27] Petersen, E. N., Pavel., M. A., Wang, H. & Hansen, S. B. Disruption of palmitate-mediated localization; a shared pathway of force and anesthetic activation of TREK-1 channels. *Biochim. Biophys. Acta Biomembr.* **1862**, 183091 (2020). 10.1016/j.bbamem.2019.183091 PMC6907892 31672538

[28] Maingret, F., Patel, A., Lazdunski, M. & Honoré, E. The endocannabinoid anandamide is a direct and selective blocker of the background K(+) channel TASK-1. *EMBO J.* **20**, 47-54 (2001). 10.1093/emboj/20.1.47 PMC140203 11226154

[29] Bautista, D. et al. Pungent agents from Szechuan peppers excite sensory neurons by inhibiting two-pore potassium channels. *Nat. Neurosci.* **11**, 772–779 (2008). 10.1038/nn.2143 PMC3072296 18568022

[30] Kollert, S., Dombert., B., Döring, F. & Wischmeyer, E. Activation of TRESK channels by the inflammatory mediator lysophosphatidic acid balances nociceptive signalling. *Sci. Rep.* **5**, 12548 (2015). 10.1038/srep12548 PMC4519772 26224542

[31] Riel, E. B. et al The versatile regulation of K2P channels by polyanionic lipids of the phosphoinositide and fatty acid metabolism. *J. Gen. Physiol.* **154**, e202112989 (2022). 10.1085/jgp.202112989 PMC8693234 34928298

[32] Lolicato M (2017). K2P2.1 (TREK-1)-activator complexes reveal a cryptic selectivity filter binding site. Nature.

[33] Pope L, Lolicato M, Minor DL (2020). Polynuclear ruthenium amines inhibit K cell. Chem. Biol..

[34] Lolicato, M. et al. K2P channel C-type gating involves asymmetric selectivity filter order-disorder transitions. *Sci. Adv.* **6**, eabc9174 (2020). 10.1126/sciadv.abc9174 PMC7608817 33127683

[35] Schrecke, S. et al. Selective regulation of human TRAAK channels by biologically active phospholipids. *Nat. Chem. Biol.* **17**, 89–95 (2021). 10.1038/s41589-020-00659-5 PMC7746637 32989299

[36] Zhang Q (2022). ‘C-type’ closed state and gating mechanisms of K2P channels revealed by conformational changes of the TREK-1 channel. J. Mol. cell Biol..

[37] Cheng, W. W. L., Arcario., M. J. & Petroff, J. T. Druggable lipid binding sites in pentameric ligand-gated ion channels and transient receptor potential channels. *Front. Physiol.* **12**, 798102 (2022). 10.3389/fphys.2021.798102 PMC8777383 35069257

[38] Hansen, S. B., Tao, X. & MacKinnon, R. Structural basis of PIP2 activation of the classical inward rectifier K+ channel Kir2.2. *Nature* **477**, 495–498 (2011). 10.1038/nature10370 PMC3324908 21874019

[39] Zheng, Y. et al. Structural insights into the lipid and ligand regulation of a human neuronal KCNQ channel. *Neuron* **110**, 237-247.e4 (2022). 10.1016/j.neuron.2021.10.029 34767770

[40] Sun, J. & MacKinnon, R. Structural basis of human KCNQ1 modulation and gating. *Cell* **180**, 340–347.e9 (2020). 10.1016/j.cell.2019.12.003 PMC7083075 31883792

[41] Niu, Y., Tao, X., Touhara, K. K. & MacKinnon, R. Cryo-EM analysis of PIP _2_ regulation in mammalian GIRK channels. *Elife* **9**, e60552 (2020). 10.7554/eLife.60552 PMC7556866 32844743

[42] Panasawatwong, A., Pipatpolkai, T. & Tucker, S. J. Transition between conformational states of the TREK-1 K2P channel promoted by interaction with PIP 2. *Biophys. J.* **121**, 2380–2388 (2022). 10.1016/j.bpj.2022.05.019 PMC9279171 35596528

[43] Chemin J (2005). A phospholipid sensor controls mechanogating of the K+ channel TREK-1. EMBO J..

[44] Murbartián, J., Lei., Q., Sando, J. J. &Bayliss, D. A. Sequential phosphorylation mediates receptor- and kinase-induced inhibition of TREK-1 background potassium channels. *J. Biol. Chem.* **280**, 30175–30184 (2005). 10.1074/jbc.M503862200 16006563

[45] García, G., Karina, M.-R. A., Oviedo, N. & Murbartián, J. PKC- and PKA-dependent phosphorylation modulates TREK-1 function in naïve and neuropathic rats. *J. Neurochem.* **157**, 2039–2054 (2021). 10.1111/jnc.15204 33006141

[46] Lenaeus, M. J., Burdette, D., Wagner, T., Focia, P. J. & Gross, A. Structures of KcsA in complex with symmetrical quaternary ammonium compounds reveal a hydrophobic binding site. *Biochemistry* **53**, 5365–5373 (2014). 10.1021/bi500525s PMC4139162 25093676

[47] Brohawn, S. G., del Mármol, J. & MacKinnon, R. Crystal structure of the human K2P TRAAK, a lipid- and mechano-sensitive K+ ion channel. *Science* **335**, 436–441 (2012). 10.1126/science.1213808 PMC3329120 22282805

[48] Aryal, P. et al. Bilayer-mediated structural transitions control mechanosensitivity of the TREK-2 K2P channel. *Structure* **25**, 708–718.e2 (2017). 10.1016/j.str.2017.03.006 PMC5415359 28392258

[49] Clausen, M., Jarerattanachat, V., Carpenter, E., Sansom, M. & Tucker, S. Asymmetric mechanosensitivity in a eukaryotic ion channel. *Proc. Natl Acad. Sci. USA* **114**, E8343–E8351 (2017). 10.1073/pnas.1708990114 PMC5635901 28923939

[50] Martinac, B. et al. Tuning ion channel mechanosensitivity by asymmetry of the transbilayer pressure profile. *Biophys. Rev.* **10**, 1377–1384 (2018). 10.1007/s12551-018-0450-3 PMC6233343 30182202

[51] Zhang Y (2021). Visualization of the mechanosensitive ion channel MscS under membrane tension. Nature.

[52] Flegler, V. J. et al. Mechanosensitive channel gating by delipidation. *Proc. Natl Acad. Sci. USA* **118**, e2107095118 (2021). 10.1073/pnas.2107095118 PMC8379960 34376558

[53] Wague, A. et al. Mechanistic insights into volatile anesthetic modulation of K2P channels. *Elife* **9**, e59839 (2020). 10.7554/eLife.59839 PMC7781597 33345771

[54] Maingret F (2000). TREK-1 is a heat-activated background K(+) channel. EMBO J..

[55] van Meer, G., Voelker, D. R. & Feigenson, G. W. Membrane lipids: where they are and how they behave. *Nat. Rev. Mol. Cell Biol.* **9**, 112–124 (2008). 10.1038/nrm2330 PMC2642958 18216768

[56] Maingret, F., Patel, A. J., Lesage, F., Lazdunski, M. & Honoré, E. Lysophospholipids open the two-pore domain mechano-gated K(+) channels TREK-1 and TRAAK. *J. Biol. Chem.* **275**, 10128–10133 (2000). 10.1074/jbc.275.14.10128 10744694

[57] Dadi, P. et al. Selective small molecule activators of TREK-2 channels stimulate dorsal root ganglion c-fiber nociceptor two-pore-domain potassium channel currents and limit calcium influx. *ACS Chem. Neurosci.* **8**, 558–568 (2017). 10.1021/acschemneuro.6b00301 PMC5901755 27805811

[58] Sandoz G, Douguet D, Chatelain F, Lazdunski M, Lesage F (2009). Extracellular acidification exerts opposite actions on TREK1 and TREK2 potassium channels via a single conserved histidine residue. Proc. Natl Acad. Sci. USA.

[59] Bockenhauer, D., Zilberberg, N. & Goldstein, S. KCNK2: reversible conversion of a hippocampal potassium leak into a voltage-dependent channel. *Nat. Neurosci.* **4**, 486–91 (2001). 10.1038/87434 11319556

[60] Ben Soussia, I. et al. Mutation of a single residue promotes gating of vertebrate and invertebrate two-pore domain potassium channels. *Nat. Commun.* **10**, 787 (2019). 10.1038/s41467-019-08710-3 PMC6377628 30770809

[61] Su, Z., Brown, E., Wang, W. & MacKinnon, R. Novel cell-free high-throughput screening method for pharmacological tools targeting K+ channels. *Proc. Natl Acad. Sci. USA* **113**, 5748–5753 (2016). 10.1073/pnas.1602815113 PMC4878532 27091997

[62] Tong, A. et al. Direct binding of phosphatidylglycerol at specific sites modulates desensitization of a ligand-gated ion channel. *eLife* **8**, e50766 (2019). 10.7554/eLife.50766 PMC6855808 31724949

[63] Marty, M. T. et al. Bayesian deconvolution of mass and ion mobility spectra: from binary interactions to polydisperse ensembles. *Anal. Chem.* **87**, 4370–4376 (2015). 10.1021/acs.analchem.5b00140 PMC4594776 25799115

[64] Bligh, E. G. & Dyer, W. J. A rapid method of total lipid extraction and purification. *Can. J. Biochem. Physiol.* **37**, 911–917 (1959). 10.1139/o59-099 13671378

[65] Suloway, C. et al. Automated molecular microscopy: the new Leginon system. *J. Struct. Biol.* **151**, 41–60 (2005). 10.1016/j.jsb.2005.03.010 15890530

[66] Zivanov, J. et al. New tools for automated high-resolution cryo-EM structure determination in RELION-3. *eLife* **7**, e42166 (2018). 10.7554/eLife.42166 PMC6250425 30412051

[67] Zheng, S. Q. et al. MotionCor2: anisotropic correction of beam-induced motion for improved cryo-electron microscopy. *Nat. Methods* **14**, 331–332 (2017). 10.1038/nmeth.4193 PMC5494038 28250466

[68] Rohou, A. & Grigorieff, N. CTFFIND4: fast and accurate defocus estimation from electron micrographs. *J. Struct. Biol.* **192**, 216–221 (2015). 10.1016/j.jsb.2015.08.008 PMC6760662 26278980

[69] Ramlaul, K., Palmer., C. M., Nakane, T. & Aylett, C. H. S. Mitigating local over-fitting during single particle reconstruction with SIDESPLITTER. *J. Struct. Biol.* **211**, 107545 (2020). 10.1016/j.jsb.2020.107545 PMC7369633 32534144

[70] Sanchez-Garcia, R. et al DeepEMhancer: a deep learning solution for cryo-EM volume post-processing. *Commun. Biol.* **4**, 874 (2021). 10.1038/s42003-021-02399-1 PMC8282847 34267316

[71] Pettersen, E. F. et al. UCSF Chimera–a visualization system for exploratory research and analysis. *J. Comput. Chem.* **25**, 1605–1612 (2004). 10.1002/jcc.20084 15264254

[72] Emsley, P., Lohkamp, B., Scott, W. G. & Cowtan, K. Features and development of Coot. *Acta Crystallogr. D Biol. Crystallogr.* **66**, 486–501 (2010). 10.1107/S0907444910007493 PMC2852313 20383002

[73] Afonine, P. V. et al. Real-space refinement in PHENIX for cryo-EM and crystallography. *Acta Crystallogr. D Struct. Biol.* **74**, 531–544 (2018). 10.1107/S2059798318006551 PMC6096492 29872004

