# Supplementary material for: Membrane phospholipids control gating of the mechanosensitive potassium leak channel TREK1
Source: Nat Commun. 2023 Feb 25;14:1077. doi: 10.1038/s41467-023-36765-w (PMC9968290; doi:10.1038/s41467-023-36765-w)
Supplement: Supplementary file 1 — Supplementary Information [file 41467_2023_36765_MOESM1_ESM.pdf]

Supplementary Information

Supplementary Figure 1

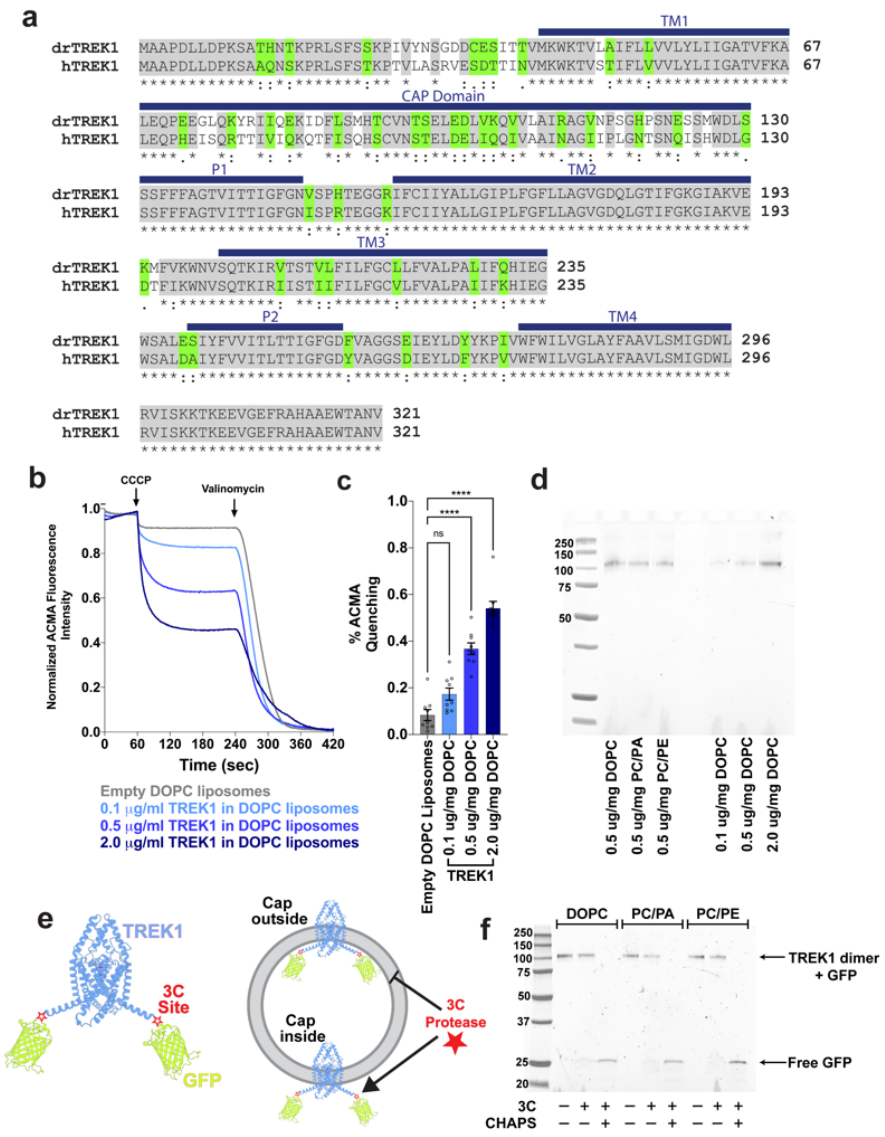

Additional characterization of the zebrafish TREK1 protein. (a) Sequence alignment of zebrafish and human TREK1 sequences. Sequence identity shown in grey, similarity in green, and non-conserved residues are left uncolored. Sequence regions corresponding to the transmembrane domains (TM), the cap domain, and the pore helices and selectivity filters (P1, P2) are noted. **(b,c).** ACMA assay studies of pure DOPC proteoliposomes containing increasing amounts of incorporated TREK1 protein (0.1 µg TREK1/mg lipid [light blue], 0.5 µg TREK1/mg lipid [medium blue], and 2.0 µg TREK1/mg lipid [dark blue]) or empty DOPC liposomes without TREK1 [grey]. Composite traces of all repeated measurements are shown in **(b)**. TREK1 dependent ACMA quenching was measured as (1 - Normalized ACMA fluorescence intensity at time t=239 s) and is shown in **(c)**. Data values for all individual measurements are shown and error bars represent mean ± SEM. For all conditions, n = 10 independent measurements derived from 2 separate proteoliposome reconstitutions. Source data are available as a source data file. Statistical analysis was performed by one way ANOVA with a Dunnett's multiple comparison test with results indicated, ns = not significant, **** = p<0.0005. **(d)** In-gel GFP fluorescence of C-terminally GFP tagged TREK1 protein after incorporation into proteoliposomes of varied lipid composition (left side of gel, pure DOPC [lane 2], DOPC + 10% POPA [lane 3], DOPC + 20% POPE [lane 4]) or varied TREK1 concentration (right side of gel, 0.1 µg TREK1/mg DOPC [lane 6], 0.5 µg TREK1/mg DOPC [lane 7], and 2.0 µg TREK1/mg DOPC [lane 8]. **(e)** Cartoon of the C-terminally GFP tagged TREK1 protein, showing the position of a 3C protease cleavage site between TREK1 and the C-terminal GFP (left). Upon incorporating this protein into proteoliposomes, only TREK1 in the "Cap inside" orientation can be cleaved by addition of 3C protease. **(f)** In-gel GFP fluorescence of the C-terminally GFP tagged TREK1 protein in proteoliposomes of varied lipid composition, as described in (d). For each lipid composition, lane 1 is untreated sample, lane 2 is a sample after 1:1 equimolar 3C treatment, and lane 3 is a sample after 3C treatment in the presence of 8mM CHAPS detergent, to solubilize the liposome.

Supplementary Figure 2

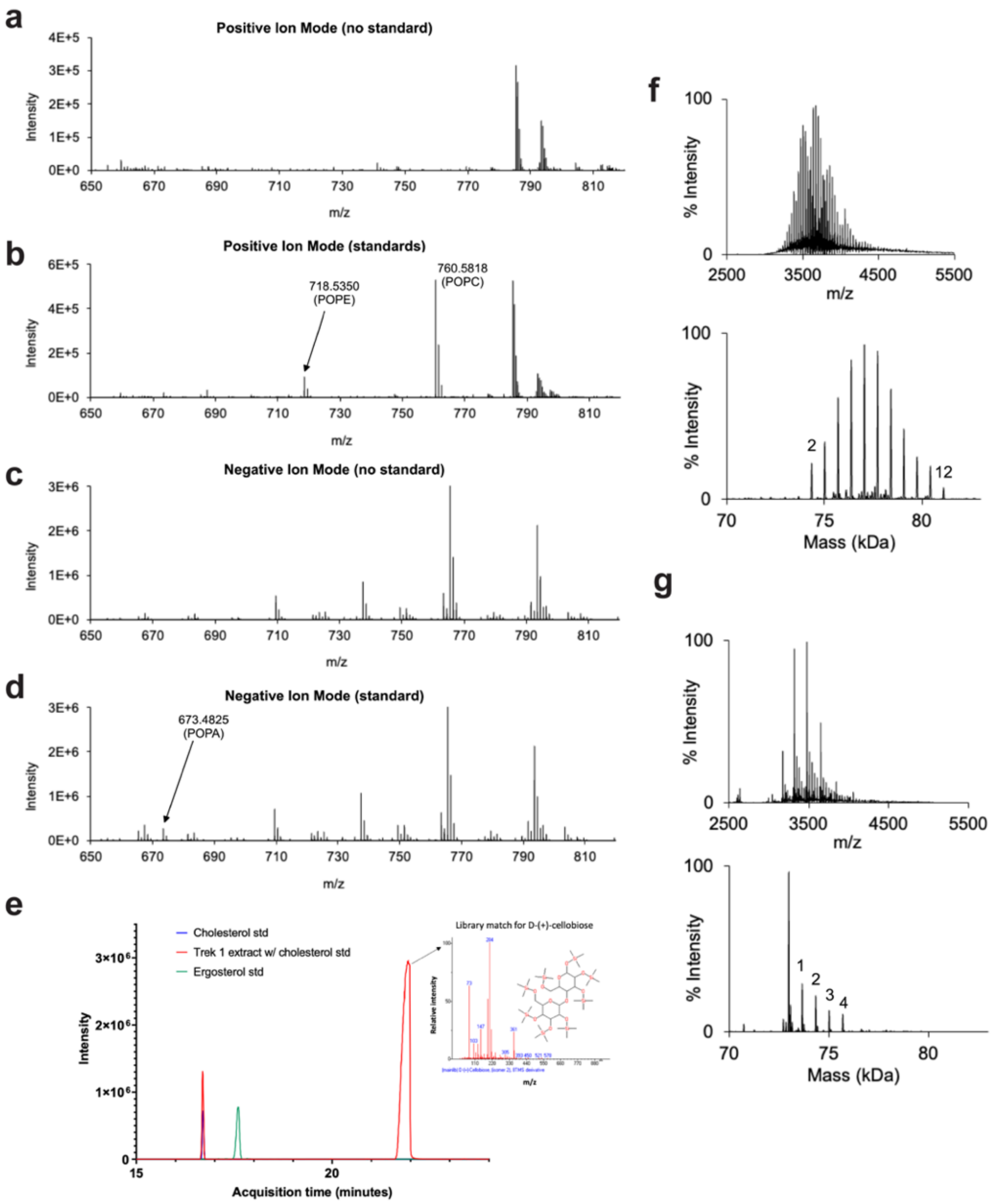

MS spectra of lipid extraction from purified TREK1. Shown are positive **(a,b)** and negative **(c,d)** ion mode spectra with and without the indicated phospholipid standards (POPC intensity = 5.7e5, POPE intensity = 1.0e5, POPA intensity = 2.4e5). In the samples without standards, POPC, POPE, or POPA were not observed. Multiple peaks other than the standards were observed in the negative and positive ion mode spectra, but none corresponded to phospholipids (see Source Data). These likely represent contaminants in the protein sample. **(e)** GC-MS selected ion monitoring of 6 ions: m/z 329, 368, and 458 (masses unique to cholesterol TMS) and m/z 337, 363, and 468 contained in the spectrum of ergosterol-TMS derivative. The red trace represents the chromatogram of the lipid extract from the TREK1 sample. The chromatogram of the pure cholesterol standard (retention time of 16.69 min) is shown in blue and the chromatogram of the ergosterol standard (retention time of 17.59) in green. The red TREK1 extract trace shows the cholesterol internal standard but ergosterol is absent. The large peak at 21.7 minutes is a disaccharide, likely D-(+)-cellobiose. **(f,g)** A second replicate of the native MS experiment shown in figure 3c. **(f)** Native mass spectrum of TREK1 dimer with POPA (upper panel) and a corresponding deconvoluted spectrum (lower panel) showing multiple peaks corresponding to TREK1 dimer with up to 12 bound POPA molecules. **(g)** Under more activating conditions, the number of bound POPA lipids is reduced to four.

Supplementary Figure 3

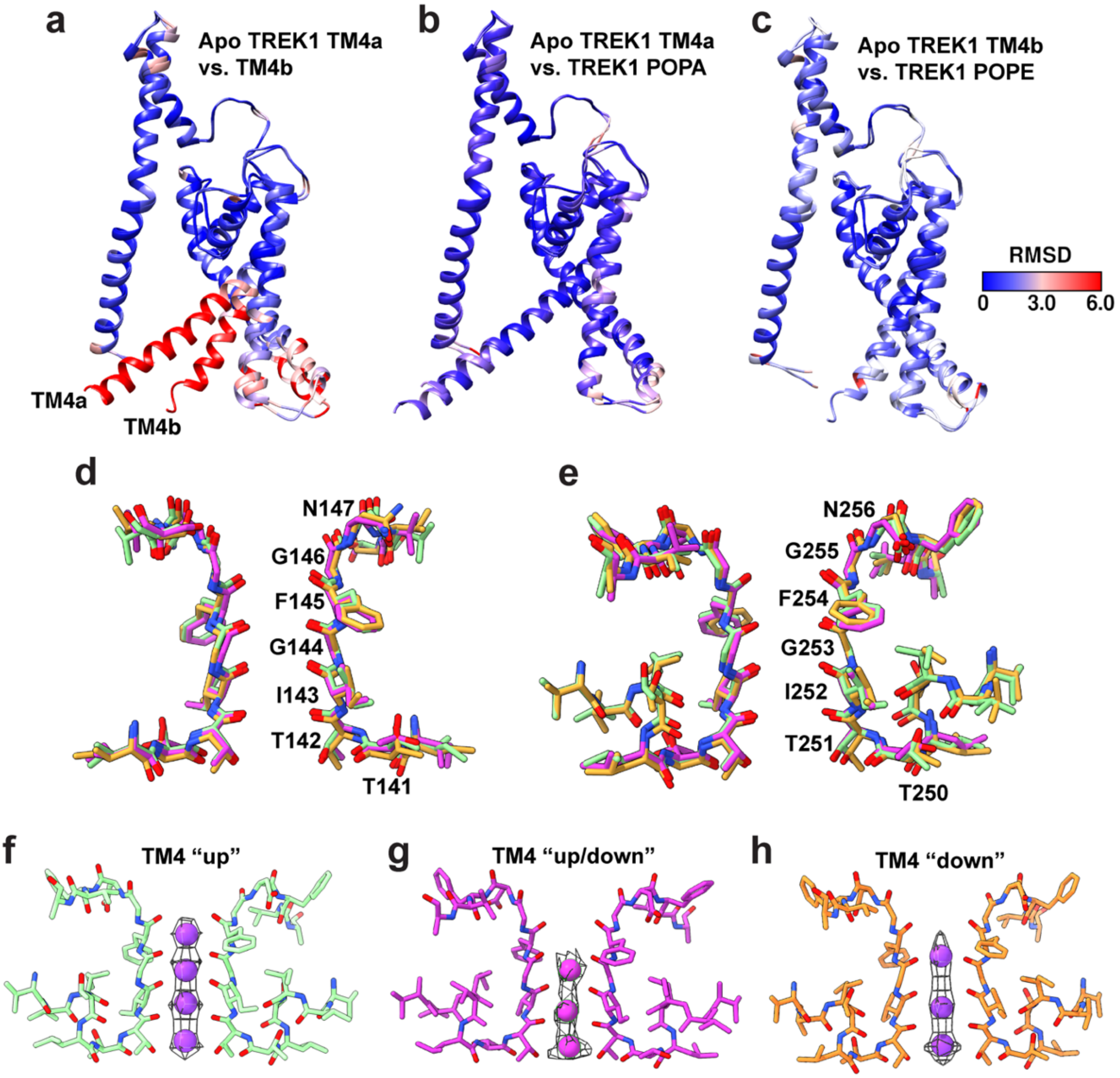

Characterization of TREK1 conformational movements. A representation of the all-atom root mean square deviation (RMSD) between **(a)** the two asymmetric subunits, TM4a and TM4b, of the apo TREK1 cryo-EM structure, **(b)** the TREK1 apo TM4a subunit with a matched subunit from the TREK1 POPA structure, both in the TM4 "up" conformation, and (c) the TREK1 apo TM4b subunit with a matched subunit from the TREK1 POPE structure, both in the TM4 "down" conformation. **(d,e)** Aligned and overlaid representations of the selectivity filter structures from the TREK1 apo (purple), TREK1 POPA (yellow) and TREK1 POPE (orange) structures. Amino acid sequences for SF1 **(d)** and SF2 **(e)** are displayed. **(f,g,h)** Cryo-EM density and ion occupancy modeled within the selectivity filters for the **(f)** TREK1 POPA structure, **(g)** TREK1 apo structure, **(h)** and the TREK1 POPE structure.

Supplementary Figure 4

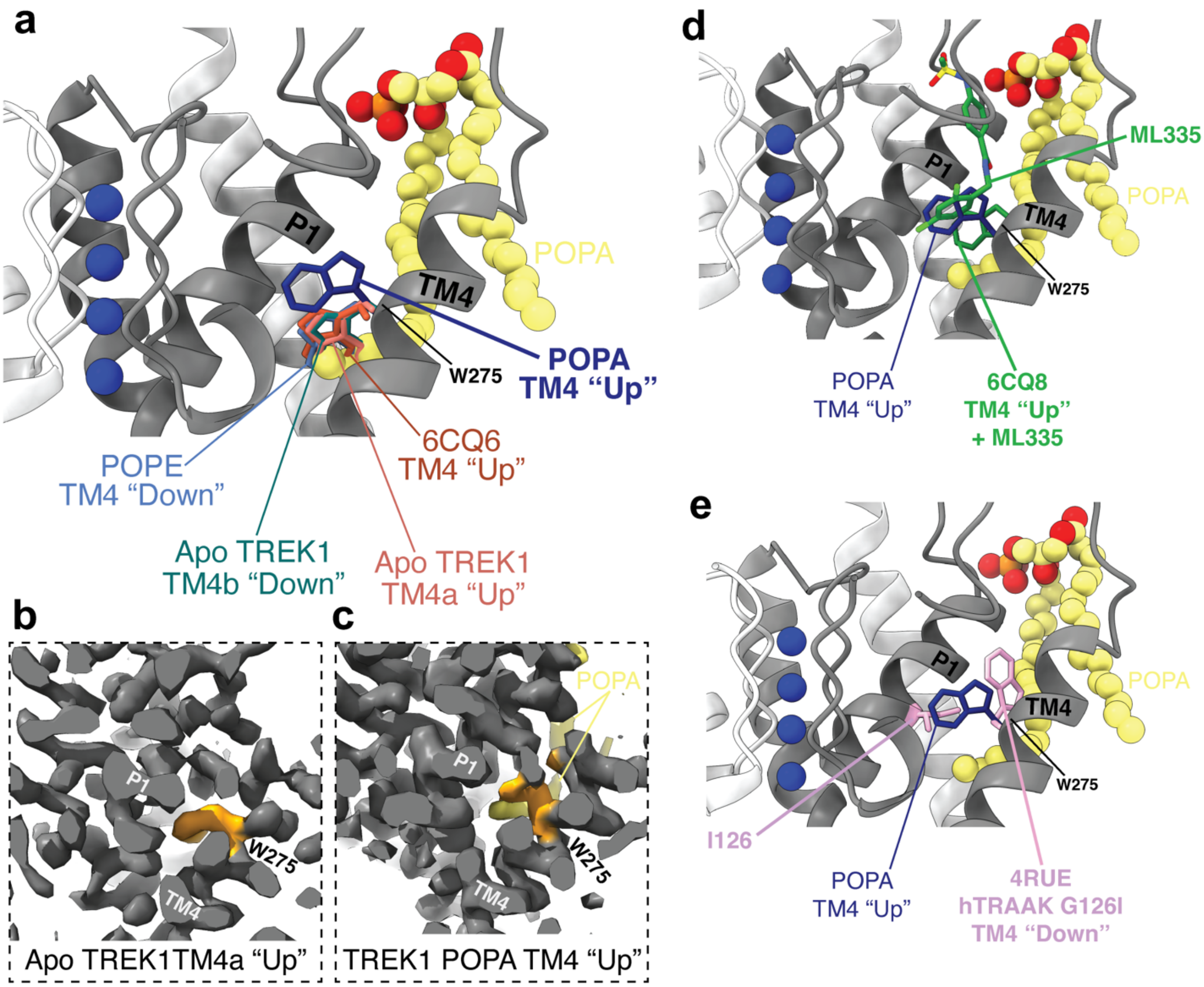

Lipid induced movement of a key residue within the POPA upper lipid binding site. Cartoon representations of the upper lipid binding site from the TREK1 POPA structure, with the positions of the W275 sidechain from numerous other structures overlaid. **(a)** The W275 residue faces outward in structures of TREK1 in the TM4 "down" (TREK1 POPE, apo TREK1 TM4b) and TM4 "up" (apo TREK1 TM4a, PDB: 6CQ6) states. The lipid acyl chain in the TREK1 POPA structure occupies the outward facing position of the W275 sidechain and moves the W275 sidechain (navy) inward. **(b,c)** Cryo-EM density maps for the Apo TREK1 TM4a subunit **(b)** and a subunit from the TREK1 POPA structure **(c)**, showing the change in position of the W275 residue (orange) in the presence of a bound POPA lipid (yellow). When reoriented inward, W275 occupies **(d)** the structurally identified binding site for the TREK1 activator ML335 (PDB: 6CQ8, green) or **(e)** the position of the isoleucine residue in a TRAAK gain of function mutant G126I (PDB: 4RUE, pink).

Supplementary Figure 5

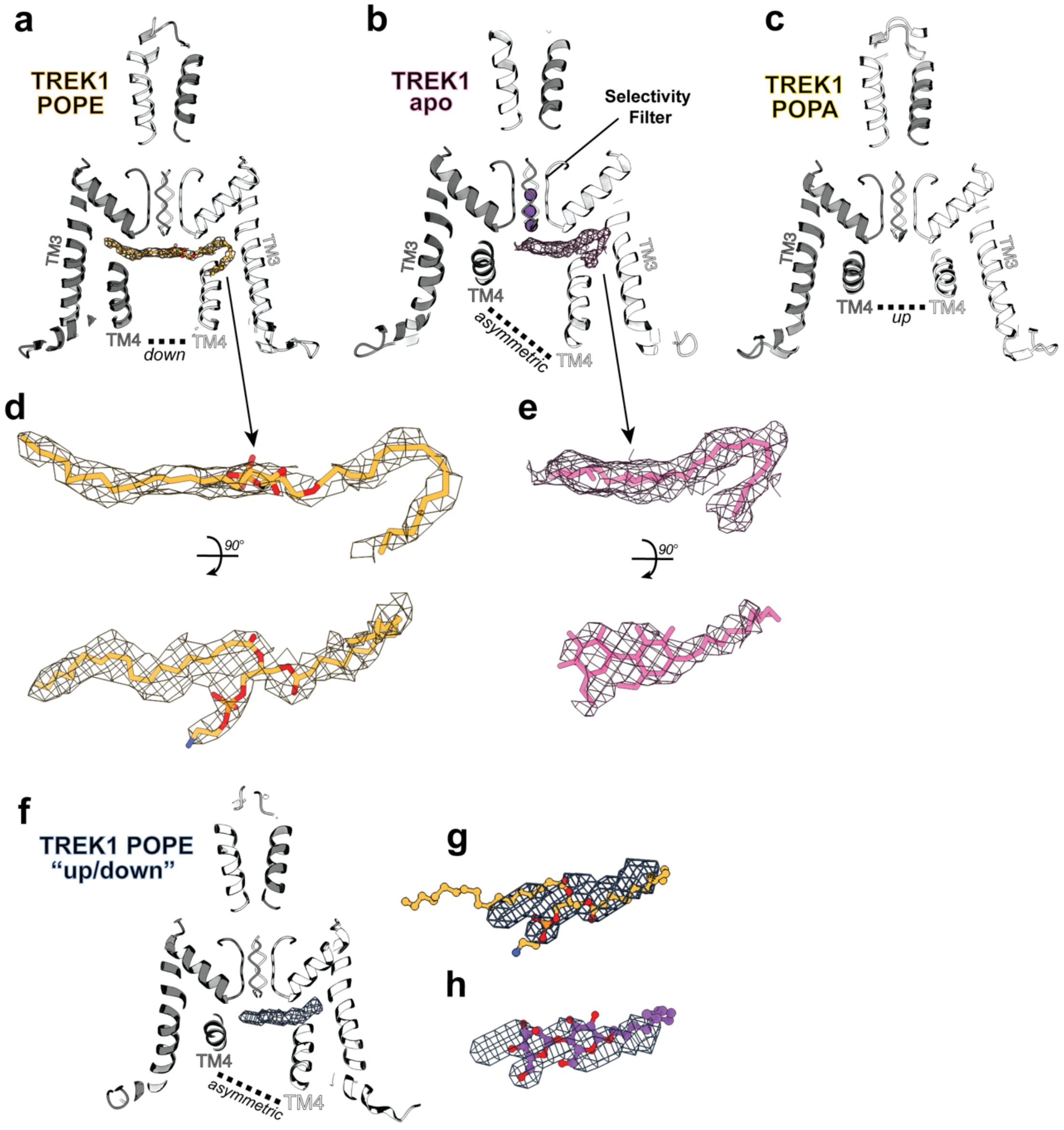

Pore blocking densities in the TREK1 structures. Structural models of TREK1, focused on the pore vestibule region for the **(a)** TREK1 POPE structure in the TM4 "down" state, due to the presence of a POPE lipid (orange) stretched out across both TM4 helices, **(b)** the apo TREK1 structure in the TM4 "asymmetric" state due to a DDM molecule blocking the upward movement of only one of the TM4, or **(c)** the TREK1 POPA structure in the TM4 "up" state with an open pore region. Note that the lipid densities in (c) are outside of the field of view of this figure and not shown. Magnified views of the cryo-EM densities for the modeled **(d)** POPE or **(e)** DDM molecules, derived from the C1 symmetrized cryo-EM maps after postprocessing in RELION. **(f)** A structural model of the less populated class in the TREK1 POPE dataset, with TM4 in the "up/down" state, showing the location of the unmodeled pore density located asymmetrically near the TM4 down helix. Magnified overlays of this density with either a POPE lipid **(g)** or a molecule of DDM detergent **(h)** are shown.

Supplementary Figure 6

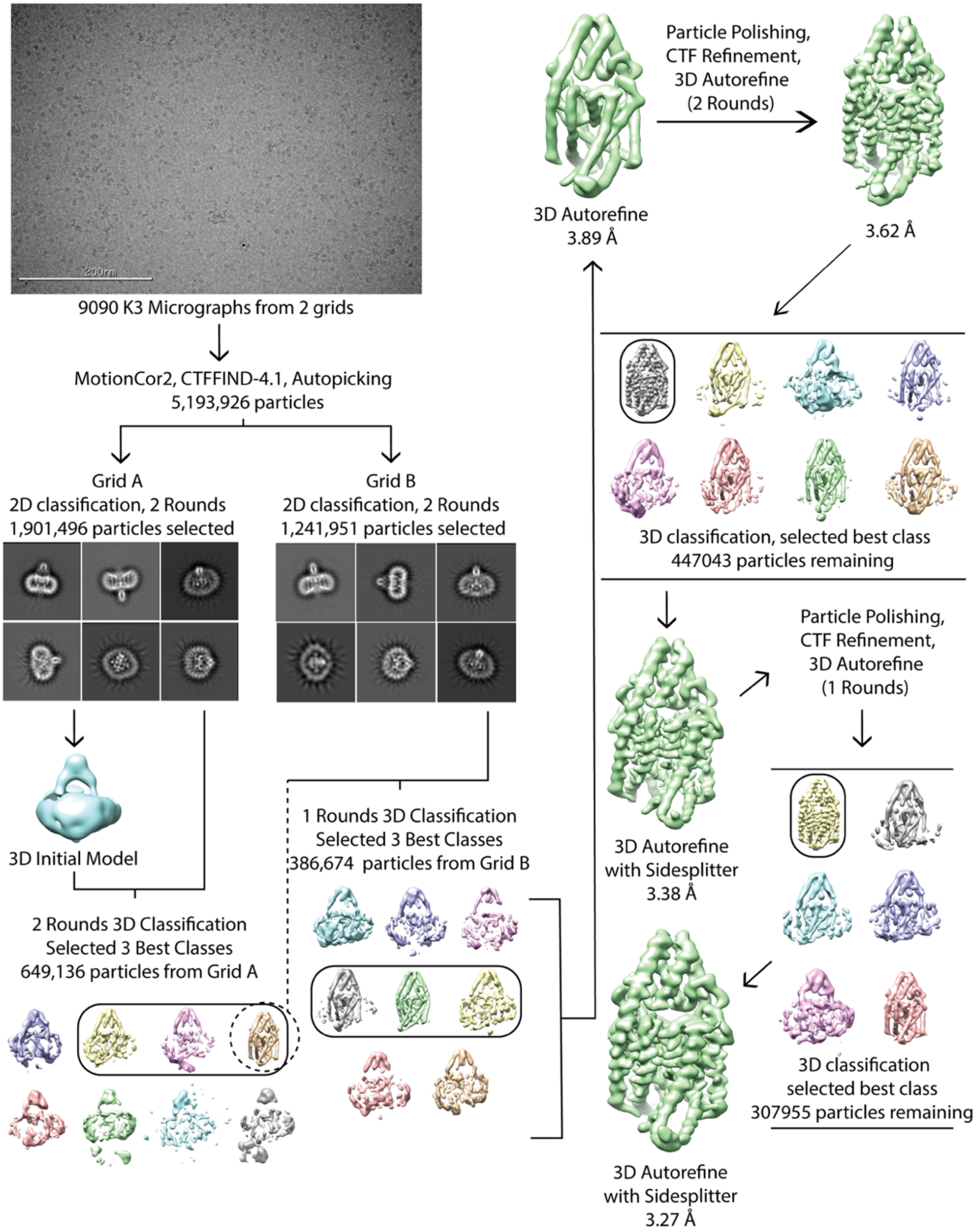

cryo-EM data processing scheme for the asymmetric TREK1 apo state. TREK1 in an asymmetric TM4 “up/down” state

Supplementary Figure 7

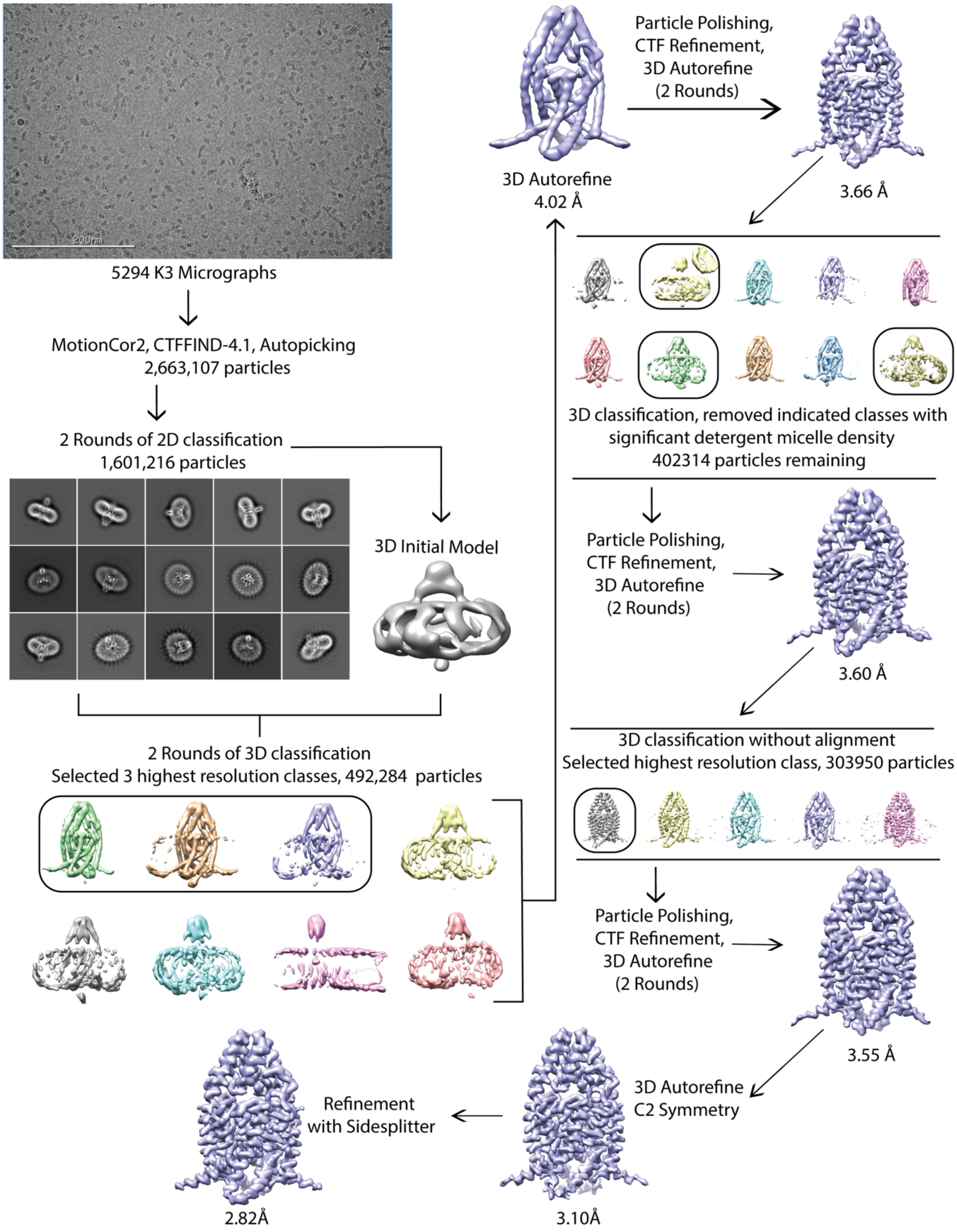

cryo-EM data processing scheme for the TREK1 POPA structure. TREK1 in the TM4 “up” state.

Supplementary Figure 8

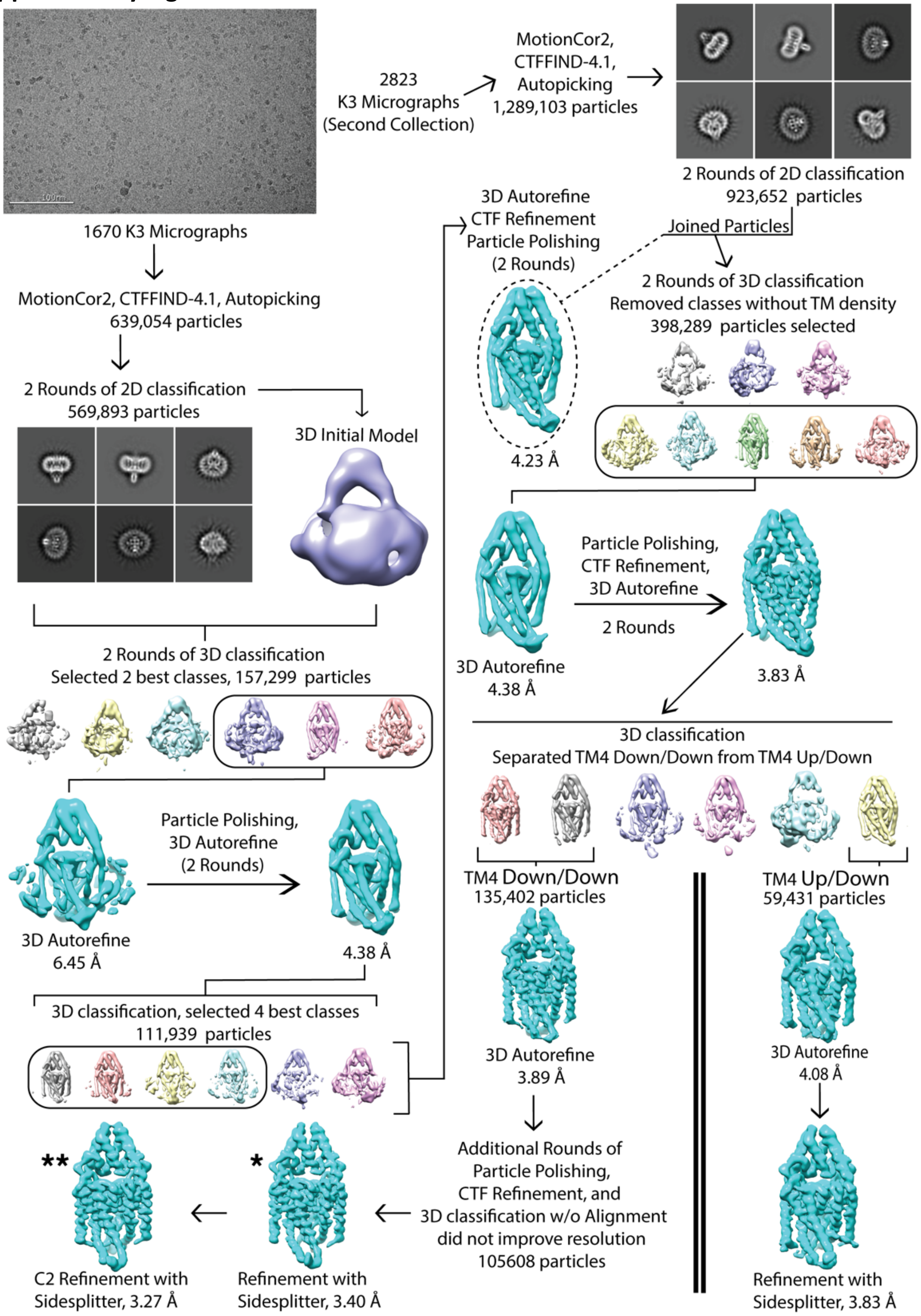

Cryo-EM data processing scheme for the TREK1 POPE structure. TREK1 in the TM4 “down” state (major group, 135k particles, left side) or TM4 “up/down” state (minor group, 59k particles, right side)**.** * denotes the final C1 refinement, used to model the POPE lipid. ** denotes the final C2 refinement, used to model the TREK1 protein structure.

Supplementary Figure 9

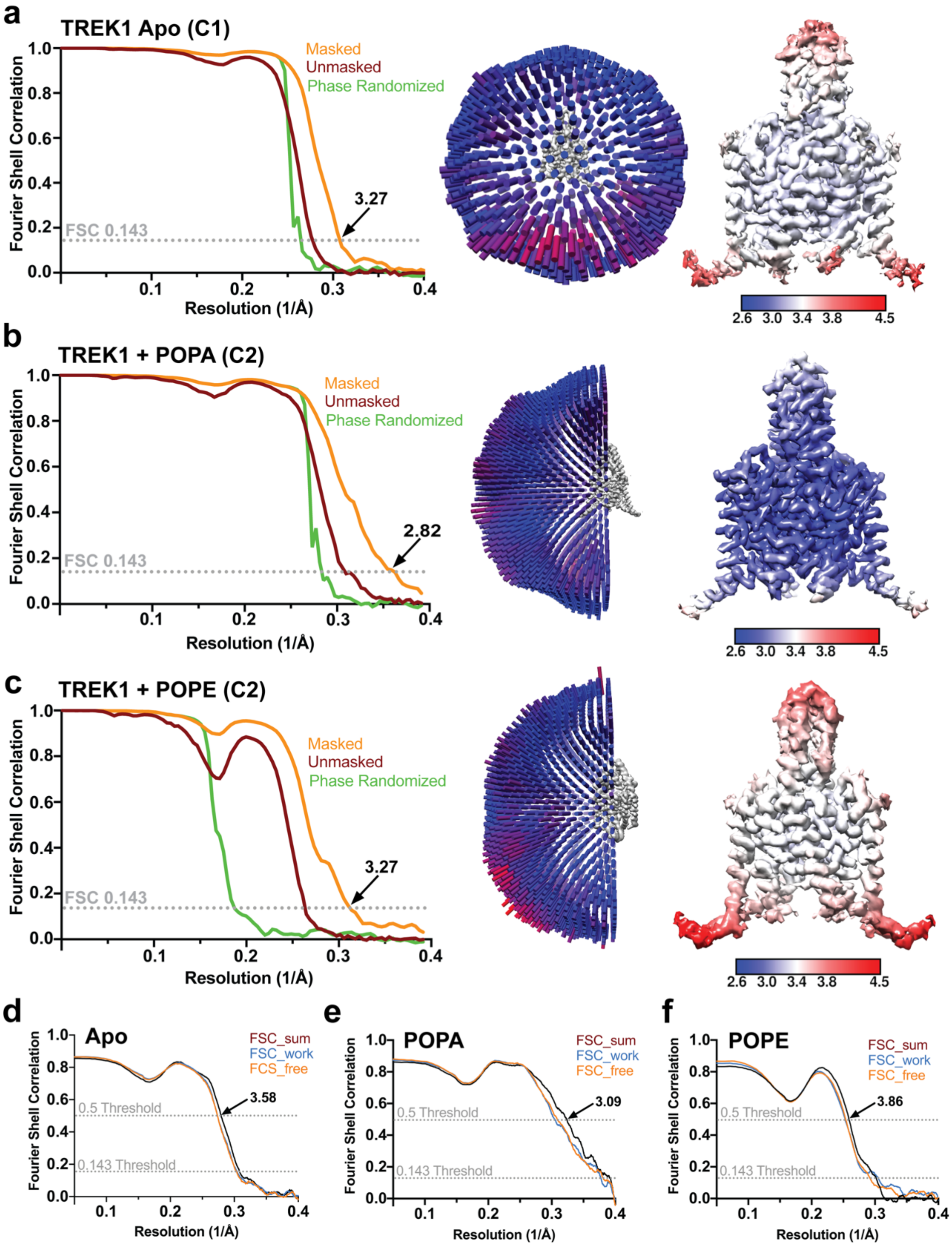

Validation of cryo-EM data. Fourier Shell Correlation (FSC) curves, angular distributions, and local resolution estimates for the **(a)** apo TREK1, **(b)** TREK1 in POPA, or **(c)** TREK1 in POPE structures. **(d,e,f)** FSC curves of refined models versus final maps for **(d)** apo TREK1, **(e)** TREK1 in POPA, and **(f)** TREK1 in POPE.

Supplementary Table 1

	Apo TREK1	TREK1 POPA	TREK1 POPE	
PDB	8DE7	8DE8	8DE9	
EMDB	27386	27387	27388	
**Data collection and processing**			Collection 1	Collection 2
Magnification	64,000X	105,000X	105,000X	
Voltage (kV)	300	300	300	
Electron exposure (e-/Å^2)	51.83	63.32	52.81	53.83
Defocus range (µm)	-0.8 to -4.0	-1.3 to -1.8	-1.3 to -2.0	
Pixel size (Å)	0.541	0.426	0.426	
Symmetry imposed	C1	C2	C2	
Initial particle images (no. post 2D)	1,035,810	492,284	398,289	
Final particle images (no.)	307,955	303,950	105,608	
Map resolution (Å) at FSC 0.143	3.27	2.82	3.27	
Map local resolution range (Å)	3.2 – 5.3	2.7 – 5.4	3.2 – 5.7	
**Refinement**				
Initial model used	6CQ6 (without ligands)	Apo TREK1	Apo TREK1	
Model resolution (Å) (at FSC 0.5)	3.58	3.09	3.86	
Map sharpening b-factor (Å^2)	-88.0957	-100.712	-85.8771	
**Model composition**				
Nonhydrogen atoms	4168	4498	4115	
Protein residues	526	552	517	
Ligands	K: 3<br>LMT: 1	K: 4<br>D21: 4	K: 3<br>PEV: 1	
**B-factors**				
Protein	35.20/237.91/127.32	27.83/128.84/62.51	37.70 /201.39/100.81	
Ligands	32.03/87.36/70.40	33.31/79.14/72.08	31.54/63.18/62.05	
**RMSD**				
Bond length (Å)	0.005	0.005	0.005	
Bond angle (°)	1.009	0.909	1.073	
**Validation**				
Molprobity score	1.47	1.43	1.60	
Clash score	8.27	5.81	6.70	
Poor rotamers (%)	0.9	0.43	0	
Ramachandran plot				
Favored (%)	97.89	97.45	96.49	
Allowed (%)	2.11	2.55	3.51	
Outliers (%)	0	0	0	

TREK1 cryo-EM collection strategy and refinement statistics. Summary table of key cryo-EM metrics including collection strategy, data processing, and model building and refinement.

Supplementary Table 2

Experimental Masses (m/z)	Intensity	Charge	Lipid	Theoretical Mass
665.4135	2.3E+05	-1		
667.429	4.5E+05	-1		
673.4835	1.6E+05	-1	PA(34:1)	673.4814
683.4241	2.2E+05	-1		
709.4755	7.4E+05	-1		
725.4701	1.9E+05	-1		
737.5072	1.1E+06	-1		
751.5222	2.8E+05	-1		
763.45	6.0E+05	-1		
765.538	3.2E+06	-1		
775.4527	4.7E+04	-1		
779.5531	1.8E+05	-1		
782.5361	8.5E+04	-1		
789.538	1.1E+05	-1		
791.554	4.1E+05	-1		
793.5699	2.1E+06	-1		
797.4381	1.6E+05	-1		
803.4818	3.4E+05	-1		
807.4579	9.0E+04	-1		
821.474	3.2E+05	-1		
835.4897	3.2E+05	-1		
849.5056	1.2E+05	-1		
879.6085	9.3E+04	-1		
907.6389	7.3E+04	-1		
935.6711	6.5E+04	-1		
1005.6786	8.6E+04	-1		
1019.6033	5.6E+07	-1		
1155.6561	4.5E+05	-1		
1196.7802	1.1E+05	-1		
611.2329	7.7E+04	1		
613.2331	2.6E+04	1		
615.2288	9.9E+03	1		
619.2272	2.3E+04	1		
621.2239	1.2E+04	1		
643.3605	9.0E+03	1		
649.2158	7.2E+03	1		
659.5457	1.5E+04	1		
687.3867	2.3E+04	1		
701.4021	8.3E+03	1		
718.5349	1.1E+05	1	PE (34:1)	718.5381
760.5820	6.0E+05	1	PC (34:1)	760.5851
785.4333	3.3E+05	2		
793.4197	9.7E+04	2		
835.4133	7.4E+04	1		

Mass Spectrometry/Lipidomic data table. Summary of results of lipidomic analysis of a DDM purified TREK1 protein sample, as shown in the spectrum of Supplemental Figure 2a-d. Experimentally added POPA, POPE, and POPC standards are denoted.
