# Supplementary material for: Membrane phospholipids control gating of the mechanosensitive potassium leak channel TREK1
Source: Nat Commun. 2023 Feb 25;14:1077. doi: 10.1038/s41467-023-36765-w (PMC9968290; doi:10.1038/s41467-023-36765-w)
Supplement: Supplementary file 2 — Reporting Summary [file 41467_2023_36765_MOESM2_ESM.pdf]

nature portfolio

Corresponding author(s): Paul M Riegelhaupt

Last updated by author(s): Jan 24, 2023

Reporting Summary

Nature Portfolio wishes to improve the reproducibility of the work that we publish. This form provides structure for consistency and transparency in reporting. For further information on Nature Portfolio policies, see our Editorial Policies and the Editorial Policy Checklist.

Statistics

For all statistical analyses, confirm that the following items are present in the figure legend, table legend, main text, or Methods section.

n/a	Confirmed	
☐	☒	The exact sample size (n) for each experimental group/condition, given as a discrete number and unit of measurement
☐	☒	A statement on whether measurements were taken from distinct samples or whether the same sample was measured repeatedly
☐	☒	The statistical test(s) used AND whether they are one- or two-sided<br>*Only common tests should be described solely by name; describe more complex techniques in the Methods section.*
☒	☐	A description of all covariates tested
☐	☒	A description of any assumptions or corrections, such as tests of normality and adjustment for multiple comparisons
☒	☐	A full description of the statistical parameters including central tendency (e.g. means) or other basic estimates (e.g. regression coefficient) AND variation (e.g. standard deviation) or associated estimates of uncertainty (e.g. confidence intervals)
☐	☒	For null hypothesis testing, the test statistic (e.g. F, t, r) with confidence intervals, effect sizes, degrees of freedom and P value noted<br>*Give P values as exact values whenever suitable.*
☒	☐	For Bayesian analysis, information on the choice of priors and Markov chain Monte Carlo settings
☒	☐	For hierarchical and complex designs, identification of the appropriate level for tests and full reporting of outcomes
☒	☐	Estimates of effect sizes (e.g. Cohen's d, Pearson's r), indicating how they were calculated

Our web collection on statistics for biologists contains articles on many of the points above.

Software and code

Policy information about availability of computer code

Data collection	Cryo-EM Data collection: Leginon 3<br>ACMA Fluorescence Quenching Data Collection: Felix GX 4.1.0<br>Mass Spectrometry Data Collection: Orbitrap ELITE 2.7, Foundation 3.1 SP4 (QExactive EMR)
Data analysis	CryoEM Data Analysis: Relion3.1.2, CTFFIND 4.1, SIDESPLITTER 1.2, DeepEMhancer, Chimera 1.14, ChimeraX 1.3, Phenix 1.19.2, Coot 0.8.9.1<br>Mass Spectrometry Data Analysis: UniDec 3.2.0, Xcalibur 4.1

For manuscripts utilizing custom algorithms or software that are central to the research but not yet described in published literature, software must be made available to editors and reviewers. We strongly encourage code deposition in a community repository (e.g. GitHub). See the Nature Portfolio guidelines for submitting code & software for further information.

Data

Policy information about availability of data

All manuscripts must include a data availability statement. This statement should provide the following information, where applicable:

- Accession codes, unique identifiers, or web links for publicly available datasets
- A description of any restrictions on data availability
- For clinical datasets or third party data, please ensure that the statement adheres to our policy

Data supporting the findings of this manuscript are available from the corresponding author upon request. A reporting summary for this article is available as a

Supplementary Information file. Cryo-EM maps of TREK1 have been deposited in the Electron Microscopy Data Bank (EMDB) under the following accession codes: TREK1 in DDM detergent: 27386 [https://www.ebi.ac.uk/emdb/EMD-27386], TREK1 in DDM/POPA mixed micelles: 27387 [https://www.ebi.ac.uk/emdb/EMD-27387], TREK1 in DDM/POPE mixed micelles 27388 [https://www.ebi.ac.uk/emdb/EMD-27388]. Atomic coordinates for all structures have been deposited in the Protein Data Bank (PDB) with accession codes 8DE7 [https://doi.org/10.2210/pdb8de7/pdb], 8DE8 [https:// doi.org/10.2210/pdb8de8/pdb], 8DE9 [https://doi.org/10.2210/pdb8de9/pdb], respectively. A crystallographically derived model of mouse apo TREK1 WT (PDB: 6CQ6 [https://doi.org/10.2210/pdb6cq6/pdb]) was used for initial model building. The source data for all ACMA fluorescence quenching results is provided as a source data file.

Human research participants

Policy information about studies involving human research participants and Sex and Gender in Research.

Reporting on sex and gender	n/a
Population characteristics	n/a
Recruitment	n/a
Ethics oversight	n/a

Note that full information on the approval of the study protocol must also be provided in the manuscript.

Field-specific reporting

Please select the one below that is the best fit for your research. If you are not sure, read the appropriate sections before making your selection.

☒ Life sciences ☐ Behavioural & social sciences ☐ Ecological, evolutionary & environmental sciences

For a reference copy of the document with all sections, see nature.com/documents/nr-reporting-summary-flat.pdf

Life sciences study design

All studies must disclose on these points even when the disclosure is negative.

Sample size	For MS studies, experimental runs were performed in duplicate on a single sample. For EM, single samples were used to collect data. In both cases, this small sample size is due to the extensive work required to generate the experimental samples.
Data exclusions	no data was excluded from analysis
Replication	Replicates for structural studies were not performed, due to the extensive work and cost required to generate experimental samples. All ACMA functional studies were replicated on N>2 independently reconstituted samples, with n>3 independent experimental repeats per reconstitution
Randomization	Randomization is not applicable for these biophysical studies, as there is no control group to randomize against.
Blinding	For MS and structural studies, blinding is not applicable as there is no control group to compare to. For ACMA assay studies, blinding was not performed, as the differing lipid compositions are easily discernible from one another by their appearance up until the extrusion step that immediately precedes measurement.

Reporting for specific materials, systems and methods

We require information from authors about some types of materials, experimental systems and methods used in many studies. Here, indicate whether each material, system or method listed is relevant to your study. If you are not sure if a list item applies to your research, read the appropriate section before selecting a response.

Materials & experimental systems

n/a	Involved in the study	
☒	☐	Antibodies
☐	☒	Eukaryotic cell lines
☒	☐	Palaeontology and archaeology
☒	☐	Animals and other organisms
☒	☐	Clinical data
☒	☐	Dual use research of concern

Methods

n/a	Involved in the study	
☒	☐	ChIP-seq
☒	☐	Flow cytometry
☒	☐	MRI-based neuroimaging

Eukaryotic cell lines

Policy information about cell lines and Sex and Gender in Research

Cell line source(s)	Pichia pastoris SMD1168H cell line used to express drTREK1 protein (source: ThermoFisher)
Authentication	Purchased commercially (with authentication documentation provided by vendor), not authenticated by our group
Mycoplasma contamination	n/a
Commonly misidentified lines (See ICLAC register)	*Name any commonly misidentified cell lines used in the study and provide a rationale for their use.*
